# An overview of Cdk1-controlled targets and processes

**DOI:** 10.1186/1747-1028-5-11

**Published:** 2010-05-13

**Authors:** Jorrit M Enserink, Richard D Kolodner

**Affiliations:** 1 Department of Molecular Biology, Institute of Medical Microbiology and Centre of Molecular Biology and Neuroscience, Oslo University Hospital, Sognsvannsveien 20, N-0027 Oslo, Norway; 2 Ludwig Institute for Cancer Research, Departments of Medicine and Cellular and Molecular Medicine, and Cancer Center, Institute for Genomic Medicine, University of California, San Diego School of Medicine, 9500 Gilman Drive, La Jolla, CA 92093-0669, USA

## Abstract

The cyclin dependent kinase Cdk1 controls the cell cycle, which is best understood in the model organism *S. cerevisiae*. Research performed during the past decade has significantly improved our understanding of the molecular machinery of the cell cycle. Approximately 75 targets of Cdk1 have been identified that control critical cell cycle events, such as DNA replication and segregation, transcriptional programs and cell morphogenesis. In this review we discuss currently known targets of Cdk1 in the budding yeast *S*. *cerevisiae *and highlight the role of Cdk1 in several crucial processes including maintenance of genome stability.

## Introduction

In eukaryotic cells, the cell cycle is controlled by cyclin dependent kinases (CDKs). Six conserved CDKs exist in the budding yeast *S*. *cerevisiae *[1-7]: Cdk1 (also known as Cdc28), Pho85 (similar to mammalian Cdk5), Kin28 (similar to mammalian Cdk7), Ssn3 (similar to mammalian Cdk8), and Ctk1 and the more recently identified Bur1 (both of which correspond to mammalian Cdk9). A single CDK, Cdk1, is necessary and sufficient to drive the cell cycle in budding yeast, but many of its functions, especially in the earlier phases of the cell cycle, are supported by the non-essential CDK Pho85, and there exists significant cross-talk between these kinases in regulation of e.g. cell morphology [8]. The other CDKs are thought to function mainly in the process of transcription [9]. In addition to the six classical CDKs, *S*. *cerevisiae *has a distant, highly diverged CDK family member, Cak1, which is involved in activation of several CDKs [10].

Budding yeast Cdk1 was first identified in a landmark genetic screen for genes that control the cell cycle performed by Hartwell [11,12]. It is a proline-directed kinase that preferentially phosphorylates the consensus sequence S/T-P-x-K/R (where × is any amino acid), although it also phosphorylates the minimal consensus sequence S/T-P [13], and recent work indicates that at least *in vitro *Cdk1 can also phosphorylate non-SP/TP sites [14-16]. Cdk1 substrates frequently contain multiple phosphorylation sites that are clustered in regions of intrinsic disorder, and their exact position in the protein is often poorly conserved in evolution, indicating that precise positioning of phosphorylation is not required for regulation of the substrate [17-19]. Cdk1 interacts with nine different cyclins throughout the cell cycle. The interaction with cyclins is important for activation of its kinase activity and also for recruitment and selection of substrates. For example, several cyclins contain a hydrophobic patch that binds the RXL (also known as Cy) motif in Cdk1 substrates. This hydrophobic patch is important for substrate selection of some cyclin-Cdk1 complexes, like e.g. Clb5-Cdk1, while for other cyclins it helps determine the cellular localization of the cyclin-Cdk1 complex, like e.g. Clb2-Cdk1 [20]. Significant overlap exists between substrates that are phosphorylated by the various cyclin-Cdk1 complexes [21], because overexpression of a single Clb (e.g. Clb1 [22] or Clb6 [23]) can rescue the lethality of a *clb1,2,3,4,5,6Δ *mutant. However, robust cell cycle progression depends on the orderly expression of cyclins [21,24-27], indicating that different cyclin-Cdk1 complexes are important for phosphorylation of the right proteins at the right time.

The fact that aberrant CDK activity underpins proliferation of tumor cells makes it a highly significant research subject [28]. Approximately 75 *bona fide in vivo *Cdk1 targets have been identified thus far (see additional Table 1). However, this number is likely to be an underestimate, because a recent study that combined specific chemical inhibition of Cdk1 with quantitative mass spectrometry identified over 300 potential Cdk1 targets [17]. In this review we discuss some of the key cell cycle processes from the perspective of Cdk1. Because it is impossible to discuss all these processes and targets in detail, we will emphasize just a few of them, while discussing the others in broader terms and referring the reader to recently published reviews and articles for further reading.

### Regulation of Cdk1

The upstream regulation of Cdk1 has been extensively reviewed [21,29-31] and therefore we will just give a more general summary of what is known about regulation of Cdk1 in budding yeast. Cyclins and CDKs are well conserved between *S*. *cerevisiae *and mammals. For instance, human cyclins can substitute for budding yeast cyclins [32], and human Cdc2 (Cdk1 in *S*. *cerevisiae*) can substitute for Cdc2 in *S*. *pombe *[33] and for Cdk1 in *S. cerevisiae *[34], illustrating the evolutionary conservation of cell cycle control. Cdk1 is inactive during G1 due to low concentrations of cyclins and the presence of the cyclin dependent kinase inhibitors (CKIs) Sic1 and Far1 [23,35]. Its activity increases at late G1, when cyclin concentrations rise and the CKIs are degraded [29]. Cdk1 activity stays high until anaphase, when it drops because cyclins are destroyed and CKIs are re-expressed [23,36]. This drop in Cdk1 activity is paramount to exit from mitosis (see section 'Cdk1 and exit from mitosis'**) **and it resets the cell cycle to a basic G1 state of low Cdk1 activity. As will be discussed later, the fluctuation in Cdk1 activity serves important functions in restricting DNA replication, repair and segregation to specific phases of the cell cycle and ensures irreversibility of the various phases of the cell cycle. The most important Cdk1 regulators are discussed below, although many more proteins can affect Cdk1 activity to a certain extent [29].

#### Cak1

The crystal structures of human Cdk2 and the cyclinA-Cdk2 complex have revealed important insights in regulation of CDK activity [37,38]. CDKs, like other protein kinases, have a two-lobed structure. CDKs are completely inactive in the absence of cyclins because (i) their active site is blocked by the T-loop, a large, flexible loop that rises from the C-terminal lobe, and (ii) several important amino acid side chains in the active site are not correctly positioned such that the phosphates of the ATP are poorly oriented for the kinase reaction. Many kinases autophosphorylate a site in their T-loop to relieve their inhibition, but not CDKs. Instead, phosphorylation of the T-loop is carried out by cyclin dependent kinase activating kinases (CAKs). Cak1, the *S. cerevisiae *CAK, is an unusual kinase that lacks many of the common features of other members of the protein kinase superfamily [39] and that bears little homology to vertebrate CAK [40]. It phosphorylates Cdk1 on T169 located within the T-loop, which is thought to result in movement of the T-loop to expose the substrate binding region and to increase the number of contacts between Cdk1 and cyclins, thus promoting the affinity of Cdk1 for cyclins [10,40-42]. Upon cyclin binding, a highly conserved helix of the upper kinase lobe called the PSTAIRE helix directly interacts with the cyclin and moves inward, causing reorientation of residues that interact with the phosphates of ATP. T-loop phosphorylation and cyclin binding are both required for full kinase activity. Phosphorylation levels of the T-loop fluctuate little throughout the cell cycle in *S. cerevisiae *[40,42], indicating that binding of cyclins is the main determinant of Cdk1 activity. Phosphorylation of T169 can be reversed by phosphatases Ptc2 and Ptc3, and overexpression of these phosphatases in yeast mutants harboring a temperature-sensitive *cak1 *allele results in synthetic lethality [43]. However, little is known about the physiological significance of dephosphorylation of T169 of Cdk1.

#### Cyclins

S. cerevisiae expresses nine cyclins that associate with Cdk1 throughout the cell cycle: three G1 cyclins and six B-type cyclins. The three G1 cyclins Cln1, Cln2 and Cln3 are involved in entry into S phase. Only a *cln1Δ cln2Δ cln3Δ *triple knockout is inviable, indicating that any of these cyclins can substitute for each other to pass Start [44]. Nonetheless, the three cyclins are thought to have different functions. Cln3 controls transcriptional programs and appears to function upstream of Cln1 and Cln2 because it stimulates the transcription of the *CLN1 *and *CLN2 *genes [45-50] (also see Section 'Cdk1 and transcriptional programs'), while Cln1 and Cln2 are important for spindle pole body duplication and initiation of bud morphogenesis (see sections 'Cdk1 and chromosome segregation' and 'Cdk1 and cell morphogenesis'). Transcription levels of *CLN3 *do not appear to fluctuate much during the cell cycle, in contrast to protein levels [45,51], indicating that Cln3 levels are regulated post-transcriptionally. Indeed, translation of *CLN3 *mRNA is an important regulatory mechanism for cell cycle entry [52,53]. In addition, the stability of Cln3, but also Cln1 and Cln2, is subject to post-translational modifications; Cln1,2,3 are all phosphorylated by Cln-Cdk1 complexes, targeting them for SCF-mediated destruction [54-56]. The expression of Cln3 is also controlled by Whi3, an RNA binding protein that is associated with the endoplasmic reticulum. It negatively regulates Cdk1 by binding *CLN3 *mRNA [57] and sequestering it at the ER [58], thus preventing accumulation of the nuclear Cdk1-Cln3 until late G1. Retention of Cln3-Cdk1 at the ER is also facilitated by interaction with the HSP70-related chaperones Ssa1 and Ssa2, while release of Cln3-Cdk1 is mediated by Ydj1, which induces the ATPase activity of Ssa1/2, thus releasing Cln3-Cdk1 which can then enter the nucleus and induce cell cycle entry [59].

Six B-type cyclins, Clb1-6, function after the G1 cyclins in the cell cycle. Expression of both Clb5 and Clb6 is induced during G1 phase, but while Clb5 is stable until mitosis, Clb6 is degraded at the G1/S border, and this is because Clb5 has an APC destruction box, causing it to be degraded by the APC, while Clb6 is targeted for destruction by the SCF upon phosphorylation by Cdk1 and Pho85 [60]. Clb5,6 are thought to be involved in timely initiation of S phase [23] and in preventing firing of origins of replication that have already fired [61] (also see section 'Cdk1 and DNA replication'). Furthermore, Clb5 is required for efficient DNA replication [62], while Clb6 inhibits transcription of G1 programs [63,64] (also see section 'Cdk1 and transcriptional programs'). Clb3,4 are expressed from S phase until anaphase and are involved in DNA replication, spindle assembly, and the G2/M-phase transition [29,65]. Clb1,2 are expressed during the G2-M phase of the cell cycle and destroyed at the end of M phase [29,66] and are involved in regulation of mitotic events such as spindle elongation, but e.g. also in bud morphogenesis by inducing the switch from polar to isotropic bud growth [67].

#### CKIs

The cyclin dependent kinase inhibitors (CKIs) Far1 and Sic1 are thought to bind cyclin-CDK complexes and prevent the kinase from interacting with its substrates [23,68-70]. The inhibitory domain of Sic1 has structural homology to mammalian p27^KIP1^, although Sic1 and p27^KIP1 ^lack sequence homology [71]. Far1 and Sic1 are expressed between the M-G1 and G1-S boundaries of the cell cycle, and outside of G1 they are unstable proteins. Far1 inhibits Cln-Cdk1 complexes at Start, especially in presence of pheromone [69] but also during vegetative growth [35], while Sic1 is thought to inhibit Clb-Cdk1 complexes [23]. Cells cannot enter S phase as long as these CKIs are present. Only when enough Clns have built up to raise Cln-Cdk1 activity to a certain threshold, can Cln-Cdk1 phosphorylate Sic1 and Far1 to target them for degradation; in fact, the only essential function of Cln-Cdk1 appears to be degrading Sic1, because lethality of the *cln1Δ cln2Δ cln3Δ *knockout is rescued by deletion of *SIC1 *[72]. Phosphorylation of Sic1 on at least 6 sites targets it for destruction by the SCF [73], while a single phosphorylation on Far1 (on S87) is sufficient for targeting it for degradation [74]. Sic1 is re-expressed in late M phase, contributing to exit from mitosis and resetting the cell cycle to a basic G1 state of low Cdk1 activity.

#### Swe1

Swe1 (the *S. cerevisiae *homolog of Wee1) is a tyrosine kinase that phosphorylates Cdk1 on Y19, resulting in inhibition of Cdk1 kinase activity [75]. In higher eukaryotes, an increase in phosphorylation levels of T14 and Y15 of Cdk1 (similar to Y19 in yeast) occurs upon DNA damage, which is important for cell cycle arrest [76]. However, *S. cerevisiae *cells do not target Cdk1 to arrest the cell cycle in response to DNA damage, but instead directly inhibit the processes associated with cell cycle progression (see section 'Cdk1 in maintenance of genome stability'). It appears that Swe1 has taken on a different role, i.e. it delays the cell cycle in response to actin and septin cytoskeleton stresses, and this checkpoint has been referred to as the morphogenesis checkpoint [77-80]. However, although Swe1 may not be involved in enforcing checkpoint-induced cell cycle arrest, it may still have a function in the DNA damage response, because the DNA replication checkpoint controls Swe1 levels to regulate bud morphogenesis, thus contributing to cell viability [81]. Swe1 preferentially phosphorylates Clb2-Cdk1 complexes, but it has intermediate activity on Clb3,4-Cdk1 complexes and low activity on the Clb5,6-Cdk1 complexes that act earlier in the cell cycle [24,75,82]. One explanation for the differential activity of Swe1 towards the different Clb-Cdk1 complexes is that Sic1 protects Clb5,6-Cdk1 complexes from Swe1-mediated phosphorylation during the earlier stages of the cell cycle; Sic1 is absent in later stages of the cell cycle and therefore cannot protect Clb1,2-Cdk1 from Swe1 [82].

Swe1 is stable during G1 and its expression peaks at the end of S phase, becoming unstable in G2 or M phase when it is rapidly degraded [83,84]. Both the APC and the SCF may have a function in degradation of Swe1 [85,86]. Degradation of Swe1 requires its recruitment to the septin ring at the bud neck, where it is phosphorylated by the kinases Cla4, Cdc5 and Cdk1, which target it for destruction [15,77,80,87,88]. However, cellular stresses that lead to perturbation of the actin or septin cytoskeleton activate the morphogenesis checkpoint by preventing Swe1 degradation, thereby inhibiting Cdk1 and delaying the cell cycle in G2 [80,83]. In addition, under normal growth conditions, *swe1Δ *mutants have a reduced cell size [84,89], and therefore Swe1 may be part of a network that monitors cell size, delaying the cell cycle until the bud has reached a critical size [84,90].

#### Mih1

The Swe1-mediated inhibitory phosphorylation of Y19 of Cdk1 is reversed by the tyrosine phosphatase Mih1 (Cdc25 in *S. pombe *and higher eukaryotes) to promote entry into mitosis [91]. Deletion of Mih1 results in increased cell size and a delay in entry into mitosis [92]. Compared to Swe1, relatively little is known about regulation of Mih1. It was recently shown that it is hyperphosphorylated in an early stage of the cell cycle and dephosphorylated as cells enter mitosis [92]. CK1 (formerly known as casein kinase 1) is responsible for most of the hyperphosphorylation of Mih1 [92]. In addition, Cdk1 directly phosphorylates Mih1, but Cdk1 activity is also required to initiate Mih1 dephosphorylation as cells enter mitosis. The consequences of these phosphorylations remain unclear [92], but it is tempting to speculate that dephosphorylation of Mih1 stimulates its phosphatase activity towards phosphorylated Y19 of Cdk1, since Mih1 dephosphorylation coincides with entry into mitosis, an event that is dependent on Cdk1 activity.

#### Cks1

Cks1 was originally identified as a high-copy suppressor of temperature sensitive *cdc28-4*, *cdc28-9 *and *cdc28-13 *mutations [93]. Cks1 likely has an important cellular function because *cks1Δ *mutants are either very sick or not viable [93,94]. Exactly what that function is has remained enigmatic [95], although recent studies have shown that it has a role in transcription by recruiting the proteasome to promoter regions [96], especially to the promoter of the essential APC component *CDC20 *[96]. Furthermore, Cks1 is required for certain proteasome functions during M-phase-specific proteolysis [97] and it increases the activity of Cln-Cdk1 complexes to promote progression through G1 phase [98].

#### Acetylation

The importance of regulation of protein function by acetylation was recognized almost 40 years ago [99], and protein acetylation is now known to regulate many diverse functions, including DNA recognition, protein-protein interaction and protein stability [100]. Interestingly, Cdk1 was recently found to be acetylated on K40, which is located within the kinase domain and which is conserved in Cdc2 (the human form of Cdk1) [101]. Mutation of this lysine residue to arginine resulted in lethality, showing that acetylation of K40 is critical for the function of Cdk1 [101]. The acetyl transferase that acetylates Cdk1 remains unknown. A good candidate could be Gcn5, which acetylates human Cdk9 on a similarly positioned lysine residue to regulate its activity [102]. However, a *gcn5Δ *mutant is viable, while a *cdc28-K40R *mutant is not, and therefore additional acetyl transferases must exist that can acetylate Cdk1.

#### Cdc14

Cdc14 is a phosphatase that is stored in the nucleolus during most of the cell cycle, but it is released during late mitosis to promote mitotic exit by dephosphorylating targets of Cdk1. This contributes to resetting of the cell cycle to a basic G1 state of low Cdk1 activity and hypophosphorylated Cdk1 targets. Regulation of Cdc14 will be discussed in more detail in section 'Cdk1 and exit from mitosis'.

## Processes and targets controlled by Cdk1

### Cdk1 and transcriptional programs

**Figure 1:**
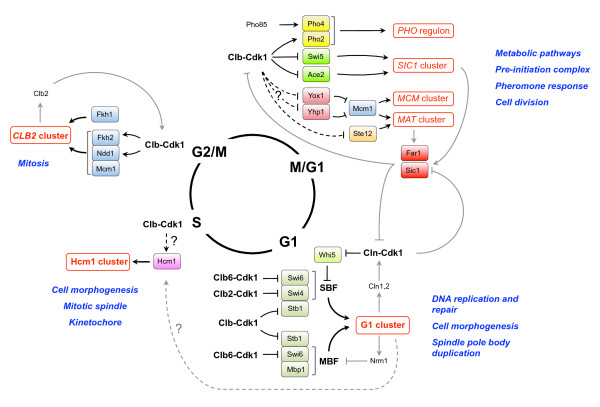
**Regulation of transcriptional programs by Cdk1 during the cell cycle**. Cdk1 is involved in positive and negative feedback loops that regulate transcriptional programs to control cell cycle progression. See text for details.

Unidirectional movement through the cell cycle is critical for cell viability and well-being of the organism; reversal of the direction of the cell cycle can have devastating consequences for the cell, including genome instability. Therefore, cells have developed mechanisms that ensure that the cell cycle is irreversible. One major mechanism that promotes unidirectionality involves regulation of distinct transcriptional programs during the different phases of the cell cycle. Typically, each transcriptional program leads to expression of sets of proteins that carry out processes important for the next phase of the cell cycle, thereby promoting unidirectional movement through the cell cycle. Furthermore, as we will discuss below, feedback mechanisms have evolved that ensure that the cell cycle is irreversible; positive feedback loops make sure that cell cycle entry is robust and switch-like, while negative feedback loops inhibit transcriptional programs to prevent reversal of the cell cycle [103-105]. Regulation of the cell cycle's transcriptional programs is highly complex, and here we focus mainly on the Cdk1-dependent aspects of transcriptional regulation (Fig. 1; for a recent review see [106]).

Under physiological conditions, activation of transcription in G1 phase is primarily carried out by Cln3-Cdk1 complexes [45-47], although in absence of Cln3, either Cln1 or Cln2 is sufficient to induce Cdk1-dependent transcription [48-50]. Approximately 200 genes are specifically expressed in G1, and together they are referred to as the G1 cluster [107,108]. Two complexes exist that mediate expression of the G1 cluster: MBF (Mlu1-box binding factor), a complex between Mbp1 and Swi6, which binds promoters harboring the MCB (Mlu1 cell cycle box) promoter element; and SBF, a complex between Swi4 and Swi6, which binds promoters harboring the SCB element (Swi4/6 cell cycle box). Although there is overlap between the classes of genes that are controlled by MBF and SBF, it appears that MBF preferentially induces transcription of genes involved in control or execution of DNA replication and repair (such as *POL2, CDC2, RNR1, CLB5 *and *CLB6*), while SBF regulates transcription of genes involved in cell cycle progression, cell morphogenesis and spindle pole body duplication (e.g. *CLN1, CLN2, PCL1, PCL2, GIN4, FKS1 *and *FKS2*) [106]. Recruitment of RNA polymerase II to the promoter region of these genes depends on Cdk1 activity [109]. Furthermore, Cln3-Cdk1-induced cell cycle entry is dependent on Swi6 (which is shared by both MBF and SBF and which mediates transcriptional activation) [110], suggesting that Cdk1 controls SBF/MBF. Indeed, Cdk1 controls SBF/MBF in multiple ways. During early G1, promoter-bound SBF is kept inactive by Whi5 [111,112]. In addition, Whi5 recruits the histone deacetylases Hos3 and Rpd3, thus further contributing to repression of transcription of G1 genes [113,114]. Efficient cell cycle entry requires phosphorylation of Whi5 by the CDKs Cdk1 and Pho85, which results in dissociation of the SBF-Whi5-Hos3/Rpd3 complex, thereby allowing SBF to activate transcription of its target genes [111-114]. In addition to Whi5, Cdk1 may directly control SBF, although mutating the Cdk1 sites in Swi4 and Swi6 had little effect on timing of transcriptional activation [63,110,115] (also see below). However, combined mutation of Cdk1 sites in Whi5 and Swi6 results in cell lethality [112,116], indicating that redundancy exists in Cdk1-mediated transcriptional activation of SBF. The mechanism of Cln3-Cdk1-mediated transcriptional activation of MBF remains unknown and may involve a regulatory mechanism similar to Whi5. Interestingly, both MBF and SBF interact with Msa1, and this interaction contributes to proper timing of the G1 transcriptional program [117].

Importantly, downregulation of Whi5 by Cln3-Cdk1 complexes results in enhanced expression of Cln1 and Cln2. Cln1/2-Cdk1 complexes can also activate SBF/MBF and inhibit Whi5, thus creating a positive feedback loop in which Cln1 and Cln2 boost their own expression, which is important for robust cell cycle entry [104].

Several mechanisms have been described for switching off the G1 program as the cell enters S phase. For instance, phosphorylation of Msa1 by Cdk1 in its NLS sequence has been reported to result in its exclusion from the nucleus [118], indicating that Cdk1 may target Msa1 to help shut off the G1 transcriptional program. However, the amplitude of transcriptional activation by SBF and MBF changes little in *msa1Δ *mutants [117], indicating that Msa1 is a relatively minor player in regulation of the G1 transcriptional program, and rather functions to fine-tune the timing of gene expression. Cyclin-Cdk1 complexes may directly target SBF and MBF to shut off the G1 transcriptional program. For instance, Clb6-Cdk1-mediated phosphorylation of Swi6 S160 results in its nuclear export [63,64]. However, binding of MBF to promoters is not regulated during the G1-S transition [103], at which time Clb6 is degraded [60], indicating that phosphorylation of Swi6 by Clb6-Cdk1 plays a relatively minor role in shutting off the G1 transcriptional program. Cdk1 may also target Swi4 to shut off the G1 program, because Clb2-Cdk1 directly interacts with Swi4 [119], and this physical interaction inhibits the ability of Swi4 to bind promoters [115,120], which may be relevant to prevent expression of the G1 program during the later stages of the cell cycle when Clb2 is present. Stb1 may also be a target of Cdk1 during exit from G1. Stb1 is a protein that interacts with Swi6 to promote the activity of SBF and MBF [121-123], and phosphorylation of Stb1 by Cdk1 releases it from promoters, although it is unclear to what extent this contributes to shutting off the G1 program [121-123]. The major player in shutting off the G1 program appears to be the transcriptional repressor Nrm1, which binds and inhibits MBF complexes [103]. Nrm1 acts through negative feedback, since Nrm1 expression is mostly dependent on MBF (although SBF can also activate *NRM1*); thus, MBF activity leads to accumulation of Nrm1, which then binds and inhibits MBF to shut off the G1 program as cells enter S phase [103].

A second transcriptional wave occurs when cells make the transition from G1 to S phase, resulting in expression of genes that make up the two S phase gene clusters, i.e. the histone cluster, consisting of all nine histone genes, and the MET gene cluster. Furthermore, it was recently discovered that a cluster of approximately 180 genes is induced during late S phase, nearly half of which function in chromosome organization and spindle dynamics, but this cluster also contains many genes encoding transcription factors that function later in the cell cycle, such as *FKH1*, *FKH2 *and *NDD1 *(see below) [124]. This cluster is controlled by the forkhead transcription factor Hcm1 [124], and here we will refer to it as the Hcm1 cluster. Hcm1 expression itself is cell cycle regulated and peaks in late G1 [124]. *HCM1 *expression is probably controlled by SBF and MBF because it has binding sites for both complexes in its promoter [125]. Hcm1 induces the expression of Fkh1, Fkh2 and Ndd1 [124], which function in the next stage of the cell cycle, which may contribute to robust cell cycle progression; Hcm1 also induces the expression of Whi5 [124], which may provide negative feedback to prevent expression of the G1 transcriptional program outside of G1. Interestingly, constitutive expression of *HCM1 *from the *GAL1 *promoter did not completely abolish the fluctuation in the cell cycle-dependent expression of two Hcm1 targets (*WHI5 *and *NDD1*), suggesting that in addition to regulating its expression, the cell cycle may also control Hcm1 activity through post-translational modifications [124]. It is tempting to speculate that Cdk1 is responsible for this regulation, because Hcm1 contains 12 potential Cdk1 sites and it is an efficient target of Clb-Cdk1 *in vitro *[126].

From the end of S phase until nuclear division in M phase a set of approximately 35 genes, including *CDC5*, *CDC20*, *SWI5 *and *ACE2*, is expressed with similar kinetics as *CLB2*, and is therefore referred to as the *CLB2 *cluster [106-108]. The *CLB2 *cluster was found to be controlled by the transcription factor called 'SFF' (SWI Five Factor), the identity of which was later shown to be the partially redundant forkhead transcription factors Fkh1 and Fkh2 [127-129]. Simultaneous deletion of *FKH1 *and *FKH2 *uncouples transcription of the *CLB2 *cluster from the cell cycle, showing that Fkh1 and Fkh2 provide the link between the cell cycle and periodic expression of the *CLB2 *cluster [127]. Fkh2 occupies the majority of SFF sites due its interaction with the transcription factor Mcm1, which increases the affinity of Fkh2 for the SFF element about 100-fold, thus outcompeting Fkh1 (which does not interact with Mcm1). Cdk1 controls transcription of the *CLB2 *cluster in multiple ways, creating a positive feedback loop in which Clb2 promotes its own synthesis [119]. For instance, Clb-Cdk1 complexes phosphorylate Fkh2 on S683 and T697 (although additional sites may exist [130]). In addition, Clb2-Cdk1 phosphorylates residue T319 on the rate-limiting transcriptional transactivator Ndd1 [131,132]; Ndd1 activates gene transcription upon recruitment by Fkh2 [133]. Interestingly, phosphorylation of both Ndd1 and Fkh2 is thought to increase their interaction, thus stimulating transcription. Phosphorylation of Ndd1 on S85 by the polo kinase Cdc5 further enhances its transcriptional activity [134]. Phosphorylation of proteins by Cdk1 can create a docking site for polo kinases [135], and it is tempting to speculate that T319 phosphorylation of Ndd1 by Cdk1 serves as a priming site for Cdc5, which subsequently would phosphorylate S85. However, phosphorylation of Ndd1-T319 is not required for phosphorylation of Ndd1-S85 [134]. Therefore, it remains unknown how Cdc5 is recruited to the Fkh2-Ndd1 complex. The key might be Fkh2, which is required for Cdc5-mediated phosphorylation of Ndd1 and which is also a target of Cdk1 [130,134].

Four clusters of genes are expressed between M phase and G1 phase: the MCM cluster, the *SIC1 *cluster, the MAT cluster and the PHO regulon [107,108]. Expression of MCM cluster genes (including *MCM2-7*, *CDC6*, *SWI4*, and *CLN3*) is controlled by the Mcm1 transcription factor, which as mentioned above is also involved in expression of the *CLB2 *cluster when it is complexed to Fkh2. However, throughout most of the cell cycle Mcm1 also binds the homeodomain repressors Yox1 and Yhp1, and genes that contain binding sites for Yox1 and Yhp1 in their promoter (the MCM cluster genes) are repressed by the Yox1-Mcm1 and Yhp1-Mcm1 complexes [136]. Yox1 and Yhp1 are unstable proteins, and Yox1 is expressed in mid-G1 through early S, while Yhp1 is expressed later in the cell cycle [108,136]. During M-G1, when both repressors are not expressed, the promoters of the MCM cluster genes are de-repressed and transcription can occur. It is currently unknown whether Cdk1 directly controls the activity of Yox1 and Yhp1, but both proteins (especially Yox1) are efficient targets of Cdk1 *in vitro *[126]. Expression of both these proteins fluctuates during the cell cycle [108,136], and the promoter regions of both *YOX1 *and *YHP1 *contain binding sites for SBF/MBF, while the *YHP1 *promoter also contains multiple binding sites for Fkh1/2 [137], suggesting that Yox1 and Yhp1 are at least indirectly controlled by Cdk1.

Expression of the *SIC1 *cluster is controlled by the transcription factors Swi5 and Ace2, which bind the same DNA sequences *in vitro *with similar affinities and whivh regulate an overlapping set of genes *in vivo *[138,139]. However, in some cases the two proteins control distinct promoters, e.g. Swi5 activates transcription of the HO endonuclease gene whereas Ace2 does not; conversely, the *CTS1 *gene encoding endochitinase is activated by Ace2 and not by Swi5 [140]. Swi5 is negatively regulated by Cdk1, because Cdk1-mediated phosphorylation of the NLS of Swi5 results in its exclusion from the nucleus [141,142]. Presumably, when Cdk1 becomes inactivated at the end of M phase, Swi5 becomes dephosphorylated, allowing it to enter the nucleus and activate transcription of the *SIC1 *cluster. Ace2 is also phosphorylated by Cdk1 on multiple residues including in the NLS [143,144], and similar to Swi5, phosphorylation of Ace2 by Cdk1 has been suggested to result in its nuclear exclusion [143,144].

Asymmetric cell division in budding yeast yields a bigger mother and a smaller daughter, and cell cycle entry is also asymmetric; mothers cells enter the cell cycle faster than daughter cells [145-148]. Interestingly, this cell cycle delay in daughter cells may be mediated by Ace2 [149,150]. Ace2 localizes to the cytoplasm during most of the cell cycle, presumably due to phosphorylation by Clb3,4-Cdk1 [143,144]. When cells exit from mitosis, Ace2 specifically localizes to the nucleus of the daughter cell, and this asymmetric localization of Ace2 requires the activity of the Mob2-Cbk1 kinase complex [151-153]. In addition, nuclear localization of Ace2 may require dephosphorylation of its Cdk1 sites [143,144], which likely occurs when Cdk1 is downregulated during mitotic exit (see section 'Cdk1 and exit from mitosis'). In the daughter cell, Ace2 represses the transcription of *CLN3*, thus providing the daughter cell with the opportunity to properly control its cell size [149,150].

The MAT cluster is a set of genes (including *FAR1*) normally induced by mating pheromone, but which is also expressed to a certain degree during M-G1 even in absence of pheromone. The rationale for basal expression of the MAT cluster in absence of pheromone could be that cells can respond quickly to arrest the cell cycle and to initiate mating once pheromone is detected. Expression of the MAT cluster depends on the aforementioned Mcm1 as well as the transcription factor Ste12, which binds to pheromone response elements (PREs) in the upstream activating sequences of its target genes [154-157]. Cdk1 has a profound effect on restricting the pheromone response (and thereby expression of genes with PRE promoter sequences) to the G1 phase of the cell cycle, which we will discuss later (see section 'Cdk1 restricts pheromone signaling to the G1 phase of the cell cycle').

The PHO regulon is also transcribed at the M-G1 boundary [107,108] and includes genes involved in scavenging and transporting phosphate [158]. The expression of these genes might not necessarily be regulated by the cell cycle, but might rather be a result of depletion of cellular phosphate pools during the metabolic processes associated with cell duplication, thus triggering the phosphate starvation response [158,159]. Regardless, it was recently shown that Cdk1 can phosphorylate the transcription factor Pho2 on S230, resulting in increased binding of Pho2 to Pho4 [160]. The Pho2-Pho4 complex is required for activation of *PHO5*, which encodes an acid phosphatase that is secreted into the periplasmic space and scavenges phosphate by working in conjunction with high-affinity phosphate transporters [161]. Pho2 also associates with the Myb-like transcription factor Bas1 to activate genes in the pyrimidine, purine and histidine biosynthesis pathways [162]. Therefore, by activating the Pho2-Pho4 complex, Cdk1 may help replenish cellular phosphate pools and stimulate biosynthesis of basic building blocks for the next round of cell division. Pho85 and Cdk1 work together in this process, because upon phosphate starvation Pho85 phosphorylates the NLS of Pho4 resulting in nuclear import of Pho4 [163].

Several other less well characterized transcription factors exist that show cell cycle-dependent expression and that are efficient targets of Cdk1 *in vitro *[126], such as Plm2 (a putative transcription factor that is induced at Start and in response to DNA damage), Tos4 (putative transcription factor similar to Plm2; Tos4 expression peaks in G1) and Pog1 (a putative transcriptional activator that promotes recovery from pheromone-induced cell cycle arrest, presumably by relieving the repression of *CLN1 *and *CLN2 *[164]). It will interesting to see how these proteins impact the cell cycle and whether they are controlled by Cdk1.

While Cdk1 regulates many aspects of transcription throughout the cell cycle, there is evidence that transcriptional programs are executed by a free-running oscillator independently of Cdk1 [22]. Indeed, when Cdk1 was experimentally inactivated upon entry of cells into the cell cycle, about 70% of periodic genes continued to be expressed periodically and on schedule [165], and therefore Cdk1 is unlikely to be the single determinant of global periodic transcriptional programs; rather, it may fine-tune coordination of the cell cycle with periodic transcription.

Finally, in addition to controlling transcription factors, Cdk1 has also been reported to affect the process of transcription in other ways. For instance, together with Cks1 it recruits the proteasome (which enhances efficient transcription elongation by RNA polymerase II [166,167]) to the *GAL1 *ORF during galactose-induced transcription of the *GAL1 *gene to promote transcription [168]. Interestingly, this appears to be independent of its kinase activity, suggesting that Cdk1 may function as an adaptor protein [168]. Cdk1 may also modulate transcription by regulating chromatin modifiers. For example, it was recently suggested that Clb2-Cdk1 is required for NuA4-mediated acetylation of Htz1 on Lys14 [169], and Cdk1 has been speculated to exert this function through phosphorylation of Yng2 [169], which is a component of NuA4 required for histone acetyltransferase activity and which may be phosphorylated on Cdk1 sites in vivo [17]. Cdk1 may also affect histone acetylation by promoting dissociation of the repressive Sin3 histone deacetylase complex from the *CLB2 *promoter, resulting in a local, transient increase in histone H4 acetylation, which facilitates transcription [170]. The molecular target of Cdk1 in this process is not known, but could be Sin3 itself, because in proteomic studies it has been found to associate with cyclins [144] and to be phosphorylated on Cdk1 sites *in vivo *[17,171].

### Cdk1 and cell morphogenesis

**Figure 2:**
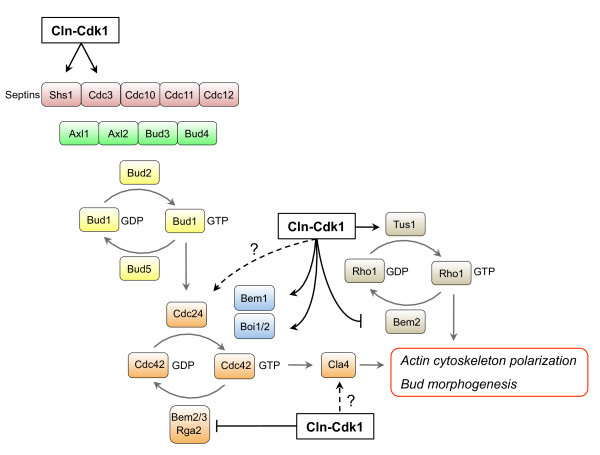
**Cdk1 and control of bud morphogenesis**. Landmark proteins select the bud site, which is followed by recruitment and activation of Bud1, which in turn recruits and activates the small GTPase Cdc42. Cdk1 reinforces activation of Cdc42 by inhibiting the activity of the GAPs Bem2/3 and Rga2, and by phosphorylating the adaptor proteins Bem1 and Boi1/2. Cdk1 may also activate Cdc42 by phosphorylating the GEF Cdc24. GTP-bound Cdc42 then recruits Cla4, which mediates polarization of the actin cytoskeleton, which is required for bud growth. In addition, Cdk1 promotes the activity of the small GTPase Rho1 by inhibiting Bem2 and by activating the GEF Tus1, which supports bud growth. The septins Shs1 and Cdc3 are also phosphorylated by Cdk1, which may affect the mobility of Cdc3, while phosphorylation of Shs1 may affect the activity of Cdk1 by negative feedback in a later stage of the cell cycle. See text for details.

Dramatic changes in cell morphology take place when cells enter the cell cycle and start to form a bud. Several steps can be distinguished in bud morphogenesis: The initial selection of the bud site, followed by polarized bud growth (also referred to as apical bud growth, i.e. localized growth at the tip of the bud), which is followed by isotropic bud growth (unlocalized bud growth such that the entire surface of the bud expands evenly), cytokinesis, and abscission to release the daughter cell. Cdk1 activity is crucial for bud formation, because in absence of all three G1 cyclins (Cln1, Cln2 and Cln3) no buds are formed [67], and Cdk1 also coordinates cell surface growth with the cell cycle [16]. Cdk1 cooperates with the CDK Pho85 to promote proper bud morphogenesis [172], and a *cln1 cln2 pcl1 pcl2 *quadruple mutant (lacking G1 cyclins for Cdk1 and Pho85) is not viable [173,174]. As we will discuss in this section, Cdk1 facilitates bud morphogenesis in multiple ways (Fig. 2).

#### Cell polarization

The first step in bud formation is selection of the incipient bud site, which does not occur randomly. Haploid *S. cerevisiae *cells display an axial budding pattern, meaning that the first bud forms adjacent to the pole where the birthmark is located, and during all subsequent rounds of the cell cycle the buds are located at the same pole. In contrast, diploid yeasts show a bipolar pattern, i.e. buds are formed at the cell pole that is opposite of the previous site of budding. In haploid cells, the incipient bud site is marked by landmark proteins such as Axl1, Axl2, Bud3 and Bud4, and their localization depends on septins [175]. In diploid cells, the incipient site is marked by Bud8, Bud9, and Rax2, and their localization is dependent on the polarisome complex, the actin cytoskeleton, and various other components [175]. The next step in bud selection is recruitment of Bud2 by the landmark proteins, both in haploid and in diploid cells. Bud2 is an exchange factor for the small Ras-like GTPase Bud1/Rsr1 (Rap1 in mammalian cells), and recruitment of Bud2 results in local activation of Bud1. In absence of Bud1 the cell can still form a bud, but at random sites. Once the bud site has been selected, the components for bud growth are assembled. A key player is Cdc24, which is recruited by Bud1, and recruitment of Cdc24 is dependent on Cdk1 activity. During G1, when Cdk1 is inactive, Cdc24 is sequestered in the nucleus by Far1. When the levels of Cln2 have sufficiently built up and the activity of Cln2-Cdc28 has reached a threshold, it phosphorylates Far1, resulting in its degradation and release of Cdc24, which exits the nucleus and localizes to the presumptive bud site [176]. Interestingly, Cdc24 is phosphorylated in a cell cycle-dependent manner and is triggered by Cdk1 [16,177,178]. While Cdk1 can efficiently phosphorylate Cdc24 *in vitro *[16], mutation of six CDK consensus sites in Cdc24 had no effect on its function *in vivo *[178]. Rather, the PAK-like kinase Cla4 is thought to be responsible for its phosphorylation, and Cla4 activity depends on Cdk1, although it is unknown whether Cdk1 directly phosphorylates Cla4 [179].

Cdc24 is an exchange factor for the small GTPase Cdc42, and clustering and activation of Cdc42 is a key step in polarization of the actin cytoskeleton, which is mediated by the downstream Cdc42 effectors Cla4, Ste20, Gic1 and Gic2 [180,181]. An SH3 domain containing protein, Bem1, acts as a scaffold for several proteins including Cdc24, Cdc42 and Cla4 [182], and clustering of these proteins is thought to provide a positive feedback loop that amplifies actin cytoskeleton polarization [183-185]. Phosphorylation of Cdc24 by Cla4 may abrogate the interaction between Bem1 and Cdc24, releasing Cdc24 from the site of polarized growth, thus restricting the extent of bud growth [178], although this hypothesis has been debated [177]. Scaffolding proteins are frequently used by cells as platforms on which several signaling pathways converge [186] and it is tempting to speculate that Bem1 may integrate cell cycle signals with bud growth. Bem1 is a good substrate for Cdk1 *in vitro *[126], and has been shown to be phosphorylated by Cdk1 on S72 *in vivo *[187]. However, this phosphorylation had no effect on bud emergence, and appeared to control vacuole homeostasis instead [187]. However, two other SH3 domain containing adaptor proteins, Boi1 and Boi2, which also bind Cdc42 to maintain cell polarity and to induce bud formation [188,189], were recently shown to be phosphorylated by Cdk1 *in vitro *and *in vivo *[16], and these phosphorylations were required for the function of Boi1 and Boi2.

Hydrolysis of GTP to GDP by Cdc42 is stimulated by the GAPs Rga1, Rga2, Bem2 and Bem3, and cycling between the GDP-bound state and the GTP-bound state is important for the function of Cdc42, since Cdc42 mutants that are locked in either the GDP-bound or the GTP-bound form display similar phenotypes [190]. Interestingly, Rga2 was recently shown to be directly phosphorylated by Cdk1 and Pho85 during G1 [16,191], which is thought to inhibit its activity, thus restricting activation of Cdc42 and preventing preliminary bud formation during G1 phase [191]. Furthermore, Bem2 and Bem3 are also phosphorylated and thereby inhibited by Cln-Cdk1 [192]. Therefore, during G1 phase, when Cdk1 is inactive, hypophosphorylated (i.e. active) Rga2, Bem2 and Bem3 keep Cdc42 in an inactive state, thus preventing cell polarization and bud formation during this phase of the cell cycle. Once the cell passes Start, Cdk1 promotes bud formation by stimulating Cdc42 activity in several ways: (i) by degrading Far1, thus releasing Cdc24 from the nucleus; (ii) by promoting the activity of Boi1 and Boi2, which help maintain a polarized state; and (iii) by inhibiting the activity of the Cdc42-GAPs Rga2, Bem2 and Bem3.

Once cell polarity is established, vesicles are transported along the actin cables towards the site of bud growth. Among other things, these vesicles mediate the transport of factors involved in cell wall synthesis, and fusion of these vesicles with the plasma membrane provides the membrane material that supports surface growth of the cell membrane. Continuous fusion of the vesicles with the cell membrane creates a demand for lipids. Since Cdk1 coordinates cell surface growth with the cell cycle [16], it might be expected that it controls synthesis of membrane lipids. Indeed, it was recently shown that Cdk1 phosphorylates and activates the triacylglycerol lipase Tgl4 [193]. Triacylglycerols serve as reservoirs for energy substrates (fatty acids) and membrane lipid precursors (diacylglycerols and fatty acids), and during early stages of the cell cycle Cdk1-induced lipolysis by Tgl4 mobilizes cell membrane precursors from lipid stores. In addition, Smp2, a transcriptional repressor that inhibits the expression of phospholipid biosynthetic genes, controls growth of nuclear membrane structures [194]. Smp2 is phosphorylated and inactivated by Cdk1 during a late stage of the cell cycle, when the mitotic spindle elongates, and inactivation of Smp2 leads to increased phospholipid synthesis [194,195]. Because *S. cerevisiae *undergoes closed mitosis (the nuclear membrane does not break down), additional phospholipids may be required to support nuclear membrane growth. Thus, Cdk1 coordinates membrane growth in at least two ways: (i) by mobilizing membrane precursors from lipid stores by phosphorylating and activating the lipase Tgl4 [193]; and (ii) by inducing the expression of genes involved in lipid synthesis by phosphorylating and inactivating the transcriptional repressor Smp2, thereby supporting nuclear membrane growth in a later stage of the cell cycle [194].

Vesicle transport is carried out by the type V myosin Myo2 and depends on the small Rab-family GTPase Sec4, which is activated by its GEF Sec2 [196,197]. The exocyst complex (which consists of Sec3, Sec5, Sec6, Sec8, Sec10, Sec15, Exo70, and Exo84 [198]) is an effector of Sec4 [199]. Sec3 and Exo70 localize to the site of bud growth, and the entire exocyst complex is formed once a vesicle arrives. The complex tethers the vesicle to the membrane until it is fused with the cell membrane by SNARE proteins [200]. Interestingly, when Cdk1 activity is inhibited, vesicles no longer arrive at the site of bud growth and the polarized localization of several factors involved in vesicle transport, such as Sec2, Sec3 and Myo2, is lost [16]. This is unlikely to be the result of failure to maintain a polarized actin cytoskeleton due to loss of phosphorylation of Boi1, Boi2 and Rga2, because Sec3 localization is independent of the actin cytoskeleton [201]. Given the central role of Cdk1 in bud morphogenesis, it seems likely that Cdk1 directly controls regulators of vesicle transport. Interestingly, several proteins involved in vesicle transport are efficient *in vitro *Cdk1 targets, such as Sec1, Sec2, Sec3 and Exo84 [126,202].

#### Cell wall synthesis and remodeling

As vesicles are delivered to the growing bud, extensive remodeling of the cell wall takes place, which requires coordinated activity of the biosynthetic pathways that synthesize cell wall material. A central player in coordination of cell polarity, vesicle transport and morphogenesis is the small GTPase Rho1. Rho1 controls a plethora of effector proteins: Sec3 (the exocyst component discussed above), Bni1, Fks1 and Fks2, Pkc1, and Skn7. Bni1 is a formin family protein that assembles the actin cables along which vesicles travel towards the site of polarized growth [203-207]; Fks1 and Fks2 are components of the β-1,3-glucan (a major component of the cell wall) synthase, essential for cell wall biosynthesis [208-210]; Skn7 is a yeast multicopy suppressor of defects in beta-glucan assembly, and regulates G1/S transition-specific and stress-induced transcription [211-213]; and Pkc1 is a protein kinase C homolog that controls a cell wall integrity signaling pathway that supports growth and integrity of proliferating cells [214-216]. Given all these functions of Rho1 in cell morphogenesis, it might be not surprising that its activity is controlled by Cdk1. Indeed, it was recently shown that Cdk1 directly controls the Rho1-GEF Tus1 [217]. In addition, Bem2, the previously mentioned GAP for Cdc42 that is negatively affected by Cdk1-mediated phosphorylation, also has GAP activity towards Rho1 [218]. Cdk1 may therefore positively affect Rho1 by increasing the activity of Tus1 while simultaneously inhibiting the activity of Bem2.

In addition to regulating proper localization of factors involved in cell wall synthesis, Cdk1 may also be more directly involved in cell wall synthesis. The activity, localization and stability of chitinases is cell cycle regulated [219-221], and *cak1-P212S *mutants, which are defective in activation of Cdk1, have thin, uneven cell walls and abnormalities in septum formation, and this phenotype can be suppressed by expression of an allele of *CDK1 *that bypasses the requirement for Cak1 [222]. Furthermore, the cell wall biogenesis of spores may also be controlled by Cdk1 [223]. Cdk1-mediated control of cell wall synthesis can be direct; for example, one of the chitin synthases, Chs2, becomes phosphorylated on Cdk1 consensus sites [224,225]. Chs2 resides at the ER during most of the cell cycle, but it is recruited to the bud neck during cytokinesis, where it deposits chitin as the actomyosin ring contracts [226,227]). Retention of Chs2 at the ER depends on phosphorylation on four Cdk1 consensus sites by mitotic Cdk1 [225], but when Cdk1 activity drops during mitotic exit (see section 'Cdk1 and exit from mitosis'), Chs2 becomes dephosphorylated, causing it to translocate from the ER to the bud neck.

Many more cell wall biogenesis proteins exist that deposit cell wall material, remodel the cell wall and modify cell wall components; this not only maintains cell wall integrity but also affects important processes such as water retention, adhesion, and virulence [221,228]. Given the complexity of bud formation, we believe that more Cdk1 targets remain to be identified that coordinate the cell cycle with cell polarization, vesicle sorting and cell wall biosynthesis.

#### The switch from polarized to isotropic bud growth

When the bud has reached sufficient length, bud growth switches from polarized to isotropic bud growth [67], and this isotropic switch requires redistribution of Cdc42 from the bud tip to the bud cortex [229]. Cdc42 redistribution is dependent on Clb2-Cdk1 and is inhibited by Swe1, but the relevant target of Clb2-Cdk1 in this process remains unknown [230]; however, Clb2-Cdk1 is known to repress transcription of the G1 cyclins [119], and Cln2-Cdk1 activity is continuously required for bud growth [16] (described above in section 'Cell polarization'). Thus, a simple model would be that Clb2-Cdk1 shuts down polar growth by turning off transcription of G1 cyclins.

Interestingly, it was recently shown that phospholipid flippases Lem3-Dnf1 and Lem3-Dnf2, which are localized to polarized sites on the plasma membrane, are important for the isotropic switch [231]. In *lem3Δ *mutants, in which the phospholipid phosphatidylethanolamine remains exposed on the outer membrane leaflet, Cdc42 remains polarized at the bud tip. Furthermore, phosphatidylethanolamine and phosphatidylserine stimulate the GAP activity of Rga1 and Rga2 on Cdc42, suggesting that a redistribution of phospholipids to the inner leaflet of the plasma membrane induces GAP-mediated scattering of Cdc42 from the apical growth site [231]. Although *in vivo *evidence is lacking, it is tempting to speculate that Cdk1 may control the activity of Dnf2, because Dnf2 is an efficient target of Cdk1 *in vitro *[126]. In addition, the kinase Fpk1, which has been proposed to regulate Lem3-Dnf2 [232], is a potential Cdk1 target *in vivo *[17]. Therefore, the concerted action of Cdk1 and flippases may be involved in the isotropic switch.

#### Organelle inheritance

In addition to delivery of vesicles to the growing bud, Myo2 has a key role in transport and positioning of organelles; e.g. it is involved in positioning of the nucleus [233] and delivery of peroxisomes, mitochondria, the Golgi and the vacuole to the bud [234-237]. Polarized localization of Myo2 and Myo2-mediated delivery of vesicles depends on Cdk1 activity, and therefore it might be expected that Cdk1 is either directly or indirectly involved in organelle inheritance. Indeed, Cdk1 has recently been implicated in inheritance of the vacuole [238]. Inheritance of the vacuole depends on the Myo2 binding adaptor protein Vac17 [239], which is directly phosphorylated by Clb-Cdk1 to enhance the interaction with Myo2, resulting in transport of the vacuole to the bud, thereby ensuring vacuole inheritance [238]. It is currently unknown whether inheritance of other organelles is similarly controlled by Cdk1-mediated phosphorylation of Myo2 adaptors, although Cdk1 phosphorylates the Myo2 adaptor Kar9 to control nuclear positioning (see section 'Cdk1 and chromosome segregation').

#### Septins

A final set of Cdk1 targets that we will discuss briefly is the septins. Septins belong to a family of structural proteins that form filaments that constitute the cytoskeleton. Septins organize into a ring-like structure at the bud neck where they play multiple roles, for example (i) in selection of the bud site [240]; (ii) in formation of a diffusion barrier between the mother cell and the bud which helps maintain cell polarity and which is also involved in cell aging [241-243]; and (iii) as a platform for signal transduction pathways that control the cell cycle [77]. Several septins including Cdc3, Cdc10 and Shs1 are targeted by the kinases Cla4 and Gin4, and these phosphorylations are thought to play a role in the assembly and dynamics of the septin ring [244-246]. In addition, Cdk1 can also phosphorylate the septins Cdc3 and Shs1 [14,247] (although the involvement of Cdk1 in direct phosphorylation of septins has been debated, and it has been argued that Pho85 rather than Cdk1 phosphorylates these septins [248]). Cln-Cdk1-mediated phosphorylation of Cdc3 is thought to have a function in disassembly of the old septin ring in G1 so that a new septin ring can be formed at the new bud site [247], while Cln-Cdk1 phosphorylation of Shs1 affects cell morphogenesis as well as recruitment of the kinase Gin4 [14], which positively controls Cdk1 activity in a later stage of the cell cycle by inhibiting the stability of Swe1 [249]. Finally, Cdk1-mediated phosphorylation of septins has implications for human health, because Cdk1 phosphorylates the septin Cdc11 in the pathogenic fungus *C*. *albicans *and this is required for hyphal morphogenesis [250], an important determinant of its virulence.

### Cdk1 restricts pheromone signaling to the G1 phase of the cell cycle

**Figure 3:**
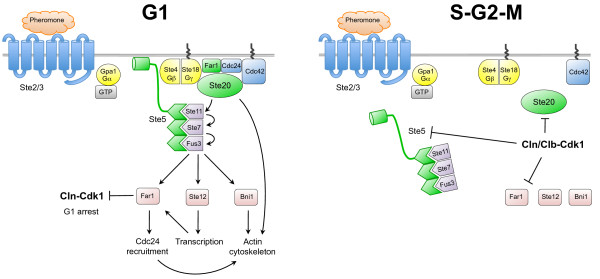
**Cdk1 restricts the pheromone response pathway to the G1 phase of the cell cycle**. (A), when pheromone is detected by the receptor during G1 phase (when Cdk1 activity is low), a signaling cascade that is mostly mediated by the βγ subunit of the heterotrimeric G protein prevents entry into S phase, polarizes the actin cytoskeleton towards the face of the cell with the highest pheromone concentration, and activates transcriptional programs. (B), binding of pheromone to the receptor outside of the G1 phase - when Cdk1 is active - does not trigger the pheromone signaling pathway because it is disconnected from its downstream components by Cdk1-mediated phosphorylation of Ste5, Ste20 and Far1. See text for details.

The *S. cerevisiae *pheromone signaling pathway is one of the best understood signaling pathways in eukaryotes (for a review see [251]). While it is believed that most essential pathway components have been identified [251], the modulation of the activity and specificity of these components during the cell cycle and during mating is less well understood; however, recent studies have identified an important role for Cdk1, which we will discuss in this section (see Fig. 3).

The pheromone response is triggered by binding of mating pheromone to the seven-transmembrane, heterotrimeric G-protein-coupled receptor (Ste2 in *MATa *cells and Ste3 in MATα cells) located on the cell surface. This induces a conformational change of the receptor, leading to GDP-to-GTP exchange by the associated G_α _subunit Gpa1, thus releasing the Ste4-Ste18 complex (the G_βγ _component of the heterotrimeric G protein) [252-257]. The Ste4-Ste18 complex, which is bound to the cell membrane because Ste18 is farnesylated and palmitoylated, recruits three effectors: (i) the Far1-Cdc24 complex, (ii) the Ste20 protein kinase, and (iii) the Ste5-Ste11 complex. Recruitment of the Far1-Cdc24 complex from the nucleus to the cell membrane results in localized activation of Cdc42 [258,259], which in turn binds and activates the PAK-like kinase Ste20 [260,261], which is membrane-bound through its interaction with Ste4-Ste18. Activation of Ste20 then results in reorganization of the actin cytoskeleton in order to form the mating projection (shmoo) that will ultimately fuse the *MATa *and *MATα *cells to form a diploid cell; reorganization of the actin cytoskeleton and subsequent shmoo growth is not unlike bud morphogenesis (discussed in section 'Cdk1 and cell morphogenesis') and makes use of similar mechanisms and components [215]. Finally, the Ste4-Ste18 complex recruits Ste5, which serves as an adaptor for the kinases Ste11 (MEKK), Ste7 (MEK) and Fus3 (MAPK). Recruitment of the Ste5 complex brings Ste11 in close proximity to Ste20, which phosphorylates and activates it [262,263]. Ste11 in turn phosphorylates Ste7, which then phosphorylates the MAP kinases Fus3 and Kss1. Both MAPKs then phosphorylate the transcription factor Ste12, which induces expression of mating type specific genes that either have a positive feedback effect (*STE2*, *FUS3*, *FAR1*) or a negative feedback effect (*SST2*, *MSG5*, *GPA1*), probably to fine-tune the pheromone response. Ste12 also activates genes involved in the process of cell fusion (e.g. *FUS1*, *FUS2*, *FIG1*, *FIG2*, *AGA1*). Targets of Fus3 include Bni1, a formin homologue the phosphorylation of which is required for actin polarization towards the site of shmoo growth [264]; Sst2, which is involved in a negative feedback loop that attenuates pheromone signaling [265]; and Tec1, which binds Ste12 to express genes required for cell differentiation, and phosphorylation by Fus3 targets it for SCF-mediated degradation, thus shifting the spectrum of Ste12-induced gene expression from differentiation genes towards pheromone response genes [266,267]. A key substrate of Fus3 is Far1, and phosphorylation of Far1 on T306 is essential for cell cycle arrest by inhibiting Cln-Cdk1 complexes [74]. It is not entirely clear how phosphorylated Far1 inhibits Cdk1 signaling, because one study found that Far1 inhibits Cln-Cdk1 kinase activity [69], while another study found that Cln-Cdk1 retains kinase activity in presence of Far1 *in vitro *[74]. One mechanism for cell cycle arrest could be that Far1 blocks access of Cln-Cdk1 to at least some of its substrates, thus inhibiting cell cycle progression.

Mating of cells should only occur during G1 phase, because this is the only period in the cell cycle when cells have a single copy of their genome (1*n*). Mating outside G1 would result in aneuploid cells with > 2*n *DNA content, which could lead to genome instability. Cdk1 is inactive during G1 phase and this permits pheromone signaling and cell mating, while outside of G1 Cdk1 is active and inhibits the mating pathway (Fig. 3A and 3B). One indication for a role for Cdk1 in regulating the pheromone response comes from the observation that in *fus3 *deletion mutants the polarized localization of Bni1, Ste20 and Ste5 upon pheromone treatment is abrogated, but this polarized localization is restored upon inhibition of Cln-Cdk1 activity, suggesting that Cdk1 negatively affects pheromone-induced polarization of cells [268]. One molecular target of Cdk1 in the negative regulation of pheromone signaling could be Ste20, which can be directly phosphorylated by Cln2-Cdk1 *in vitro *[269,270]. This is supported by the finding that mutation of all of the phosphorylation sites in Ste20 (Cdk1 consensus sites as well as non-Cdk1 sites) resulted in hypersensitivity of cells to pheromone, indicating that, under physiological levels of Cdk1 activity, phosphorylation of Ste20 negatively affects pheromone signaling [271]. However, overexpression of *CLN2 *was still able to overcome pheromone arrest in this *ste20 *phospho-site mutant [271], and therefore an additional target of Cdk1 must exist. Based on genetic data, Ste11 may also be a potential target of Cln-Cdk1 to suppress pheromone signaling [272], but it has not been demonstrated that Cdk1 actually phosphorylates Ste11. More recently, Ste5 was identified as a target of Cdk1 [273]; Cln-Cdk1 phosphorylates Ste5 on multiple residues flanking a membrane binding domain [274], which blocks membrane localization of Ste5 and its associated proteins Ste11, Ste7 and Fus3, resulting in inhibition of pheromone signaling. Furthermore, phosphoryation of Ste5 may target it for degradation by the SCF [275], further contributing to inactivation of the pheromone response pathway. It is not known whether Cdk1 phosphorylates Ste12; Ste12 controls the transcriptional program that is required for pheromone-induced cell cycle arrest and mating, and in absence of pheromone Cdk1 might be expected to inhibit Ste12 to prevent illicit expression of genes that mediate cell cycle arrest mating. Finally, Cln-Cdk1-mediated phosphorylation of the CKI Far1 on S87 targets it for degradation [74]. Presumably, destruction of Far1 results in more active Cln-Cdk1 complexes, which in a feedback loop will phosphorylate and destroy more Far1, resulting in cell cycle entry and closure of the window of opportunity for cell mating.

### Cdk1 and DNA replication

#### Initiation of DNA replication

**Figure 4:**
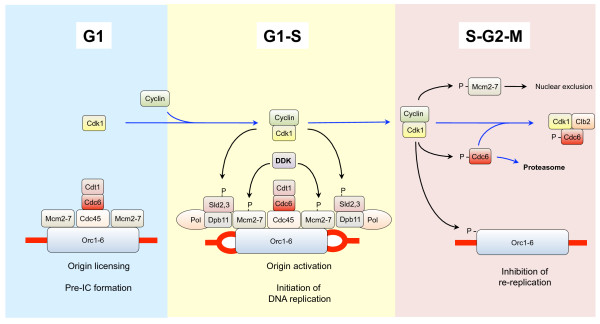
**Cdk1 and regulation of DNA replication**. During G1 phase of the cell cycle, when Cdk1 is inactive, cells assemble pre-RC complexes onto their origins of replication. When Cdk1 becomes active at the end of G1 phase it phosphorylates several components of the complex, and especially phosphorylation of Sld2 and Sld3 results in origin firing and initiation of DNA replication. After origin firing, several components dissociate and cannot re-assemble into replication-competent origins until they become dephosphorylated and Cdk1 becomes inactivated during G1, thus providing a mechanism for prevention of re-replication.

A key outcome of the cell cycle is the transmission of a complete and intact set of genetic material from one generation to the next. Two events are key to faithful execution of this process: (i) replication of the genome and (ii) segregation of the replicated genomes into the daughter cells (which we will discuss in section 'Cdk1 and chromosome segregation'). To make sure that cells do not segregate their genetic material before replication has been completed, which would result in genomic instability, these two processes are separated in time; chromosome replication occurs during S-phase while segregation of the replicated chromosomes occurs during M-phase. Cells have developed elaborate mechanisms that control both the initiation of DNA replication and that ascertain that DNA replication takes place only once per cell cycle, and Cdk1 has a central role in these events (Fig. 4, for reviews see [276-278]).

Cells prepare for DNA replication during early G1 phase, when they assemble pre-replication complexes (pre-RCs) onto their origins of replication in a process termed origin licensing, which renders the origins competent to initiate DNA synthesis [276,277]. The pre-RC is assembled onto a foundation of the six-subunit, ATP-binding Origin Recognition Complex (ORC, consisting of Orc1, Orc2, Orc3, Orc4, Orc5 and Orc6) present at replication origins [279]. ORC is involved in recruitment of the ATPase Cdc6, Cdt1 and the Mcm2-7 complex [279-281]. The Mcm2-7 complex (consisting of Mcm2, Mcm3, Mcm4, Mcm5, Mcm6 and Mcm7) functions as an ATP-dependent helicase that unwinds DNA and which is involved in both initiation of DNA replication and replication fork progression [279,280]. Mcm2-7 is recruited to the origin by ORC and Cdc6 independently of ATP hydrolysis. ATP hydrolysis by Cdc6 then stimulates the stable association of Mcm2-7 with origin DNA, after which ATP hydrolysis by ORC allows the cycle to begin again, resulting in loading of multiple Mcm2-7 complexes per origin [282,283]. Finally, a more recently identified complex called GINS associates with the Mcm2-7 helicase and is required for the initiation of chromosome replication and also for the normal progression of DNA replication forks [284].

After the pre-RCs have been assembled at the origins of replication, a transition takes place from pre-RC to pre-initiation complex (pre-IC), and this process is believed to be initiated by activation of Clb5,6-Cdk1 upon destruction of Sic1 [23,72]. A key player in pre-IC formation is Cdc45, which is recruited to the origin in a manner dependent on Clb-Cdk1 activity [285,286] and which is required for initiation of replication [287-290]. Another kinase that acts together with Cdk1 is Dbf4-dependent kinase (DDK, a dimer of the regulatory subunit Dbf4 and the kinase Cdc7), which phosphorylates the Mcm2-7 complex, resulting in recruitment of Cdc45 [286,291,292]. Cdc45 is required for recruiting DNA polymerase alpha onto chromatin, and it also associates with RPA and DNA polymerase epsilon [286]. Association of DNA polymerases alpha and epsilon with origins requires the replication protein Dpb11, a subunit of DNA polymerase epsilon holoenzyme [293].

Initiation of DNA replication follows pre-IC formation, and is induced by Cdk1-mediated phosphorylation of the proteins Sld2 and Sld3 [294-296]. Phosphorylation of Sld2 on several Cdk1 consensus sites exposes a key residue, T84, and Cdk1-mediated phosphorylation of this residue induces binding to the BRCT repeats of Dpb11 [297]. Furthermore, phosphorylation of Sld3 on T600 and S622 enhances its interaction with the BRCT repeats of Dpb11 [295]. Because Sld3 interacts with Cdc45 [298], the phosphorylation of Sld2 and Sld3 results in assembly of a complex consisting of Sld2, Sld3, Cdc45 and Dpb11 at the origin, and this constitutes the phosphorylation-dependent switch that triggers DNA replication [295,296], although the exact molecular mechanism of initiation of DNA replication by the Sld2-Sld3-Dpb11 complex still remains to be established. The requirement for Cdk1 in replication can be bypassed by expression of Sld2 containing a phosphomimetic mutation of the Cdk1 phosphorylation site *sld2-T84D *in combination with expression of a Sld3-Dpb11 chimera, and together with overexpression of Dbf4 this yields sufficient levels of DDK activity to induce DNA replication in G1 [296]. Finally, re-setting the cell for a new round of DNA replication in the next cell cycle may be mediated by the phosphatase Cdc14, which dephosphorylates DNA replication factors including Sld2, Pol12 and Dpb2 [299,300].

#### Preventing re-replication

In eukaryotic cells, DNA replication is limited to once per cell cycle because licensing only occurs in the window of low Cdk1 activity, i.e. from late mitosis through early G1 phase [276], and up-regulation of Cdk1 activity throughout the rest of the cell cycle is essential for preventing re-replication of DNA. Cdk1 targets at least three components of the pre-RC to prevent re-replication: the ORC complex, Cdc6 and the Mcm2-7 complex, and only simultaneous uncoupling of all three components from negative regulation by Cdk1 is sufficient to trigger re-replication [301]. Orc2 and Orc6 (and possibly also Orc1) are phosphorylated by Clb-Cdk1 [301], although it is not clear exactly how these modifications inhibit ORC function; this phosphorylation probably does not affect the DNA binding activity of ORC since in *S. cerevisiae *ORC remains bound to origins throughout the cell cycle [302]. Data from *Drosophila *indicate that ORC phosphorylation may inhibit the intrinsic ATPase activity of ORC [303], thus possibly interfering with loading of Mcm2-7, and a recent report showed that phosphorylation of *S. cerevisiae *Orc2 may inhibit ATP binding by Orc5, thus preventing loading of the Mcm2-7 complex [304]. Another key factor targeted by Cdk1 to prevent re-replication is Cdc6, which is only present in the cell for a limited time during the cell cycle [276,305], and several mechanisms restrict Cdc6 to G1 phase. The *CDC6 *gene is part of the MCM cluster of cell cycle regulated genes that is transcribed in late M phase, peaking at the M/G1 transition (see section 'Cdk1 and transcriptional programs'). In addition to its confined expression, Cdc6 incorporation into pre-RCs is blocked by Clb-Cdk1 so that it can no longer promote initiation of DNA replication [306]. Cdk1 directly phosphorylates Cdc6, which leads to ubiquitin-mediated proteolysis by the SCF during late G1 through S phase [307-312]. In addition, the mitotic Clb2-Cdk1 complex stably binds to Cdk1-phosphorylated Cdc6, thus preventing the binding of Cdc6 to the ORCs during M phase until Clb2 is destroyed by the APC [313]. Conversely, the interaction between Cdc6 and Clb2-Cdk1 also inhibits Cdk1 activity [314], and Cdc6 may contribute to exit from mitosis, which is triggered by inactivation of Cdk1 [314-317] (also see section 'Cdk1 and exit from mitosis'). Finally, Cdk1 targets the Mcm2-7 complex to prevent re-replication by excluding it from the nucleus outside G1 phase [318,319]. Nuclear accumulation of Mcm2-7 is dependent on two partial NLS sequences in Mcm2 and Mcm3, that when brought together form a potent NLS that targets the entire Mcm2-7 complex to the nucleus [320], and Cdk1-mediated phosphorylation of the NLS portion of Mcm3 prevents nuclear import of the Mcm2-7 complex and inhibits initiation of DNA replication [320].

Perhaps surprisingly, while checkpoints exist that arrest or slow the cell cycle during DNA damage or DNA replication stress (see section 'Cdk1 in checkpoint activation and DNA repair'), ensuring that chromosome segregation does not start until the checkpoint activating stress has been resolved [321], no mechanisms are known that monitor completion of DNA synthesis. In fact, based on the finding that *smc6-9 *mutants, which are proficient in DNA damage and replication checkpoints but fail to replicate rDNA, enter anaphase with identical kinetics as wild-type cells (despite the presence of a large amount of unreplicated rDNA), it has been suggested that cells do not monitor completion of DNA replication [322,323]. Rather, cells may simply wait a certain amount of time between onset of DNA replication and DNA segregation [323]. However, this is not likely to be an adequate explanation, because *swe1Δ *mutants, which have elevated Cdk1 activity and enter mitosis prematurely [84], do not have a <1*n *DNA content [84]. Furthermore, segregation of incompletely replicated chromosomes would result in DNA damage and chromosome instability, but in *swe1Δ *mutants neither chromosome rearrangements (which arise frequently in mutants with defects in DNA replication and repair) nor formation of Rad52 foci (which are indicative of broken DNA) are observed [324,325]. Although the possibility exists that cells indeed do not monitor completion of DNA replication, these studies indicate that it is unlikely that cells simply wait for a certain amount of time after DNA replication is finished before blindly entering mitosis.

### Cdk1 and chromosome segregation

**Figure 5:**
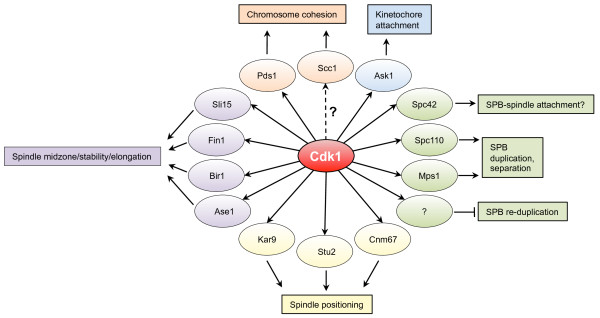
**Cdk1 controls proteins involved chromosome segregation**. Cdk1 controls chromosome cohesion by phosphorylating Pds1 and possibly the cohesin Scc1. Assembly of the mitotic spindle is also controlled by Cdk1, because it phosphorylates Spc42 and Mps1, which is important for SPB duplication, as well as Spc110, which may play a role in attachment of the SPB to the mitotic spindle. Cdk1 also prevents SPB re-duplication, but the molecular mechanism remains to be determined. Spindle positioning is mediated by Cdk1-dependent phosphorylation of Kar9, the SPB component Cnm67, and possibly Stu2. Later in the cell cycle Cdk1 phosphorylates Ase1, Bir1, Fin1 and Sli15 to modulate spindle stability and elongation.

In addition to DNA replication, a second cell cycle event is crucial for faithful transmission of genetic material from one generation to the next: segregation of the replicated genomes into the daughter cells. Successful segregation of the genetic material involves several important processes such as chromosome condensation, chromosome cohesion and dissolution, assembly of the mitotic spindle, attachment of chromosomes to the spindle, spindle elongation and separation of chromosomes, mitotic exit, and cytokinesis. As we will discuss below, Cdk1 plays important roles in several of these processes (Fig. 5).

#### Chromosome cohesion and condensation

As DNA replication takes place, an essential process termed chromosome cohesion ensures that sister chromatids are held together until anaphase. Chromosome cohesion prevents premature separation of sister chromatids and is carried out by the cohesion complex. The core of the cohesion complex is a heterodimer of Smc1 and Smc3, which binds Scc1 and Scc3 [326]. Chromosome cohesion is cell cycle regulated and several steps can be distinguished [326]: (i) loading of cohesin onto chromatin (which occurs before onset of S phase) by the Scc2-Scc4 complex; (ii) conversion of cohesin to a cohesive state (establishment of cohesion) in a manner that depends on Eco1 and which occurs concomitantly with DNA replication; and (iii) stabilization and maintenance of cohesion. Genetic studies have indicated that chromosome cohesion is at least in part dependent on *CDK1 *and that *CDK1 *may function upstream of *SCC1 *[327]. Indeed, mutations that reduce Cdk1 activity lead to chromosome cohesion defects [328,329]. The molecular target of Cdk1 in chromosome cohesion remains elusive. Eco1 is an attractive candidate because it is required for establishment for cohesion and it is a good target of Cdk1 *in vitro *[126], however mutation of the Cdk1 consensus sites in Eco1 does not affect chromosome cohesion [329]. Scc1 could also be a good candidate, because (i) Cdk1 activity appears to be required for Scc1 activity; (ii) Scc1 is a regulatory component of the cohesin complex and is a common target of several kinases that modulate chromosome cohesion including Chk1 and polo kinase [330,331]; and (iii) in *S*. pombe Rad21 (*S.p*. Scc1) is phosphorylated by Cdk1 [332], although the consequences of this phosphorylation remain unknown.

Dissolution of cohesion takes place at anaphase, when all the chromosomes are properly bi-oriented on the metaphase plate and attached to the mitotic spindle, which induces activation of the anaphase promoting complex (APC). The APC degrades a protein called securin (Pds1 in budding yeast), which is an inhibitor of separase (Esp1). Esp1 is a protease that cleaves Scc1, resulting in disruption of cohesion, which is a prerequisite for chromosome segregation [333]; thus, Pds1 functions to prevent precocious chromosome segregation during earlier stages of M phase [333]. Importantly, dissolution of chromosome cohesion is inhibited by Cdk1, because Cdk1 phosphorylates Pds1, thus protecting it from APC-mediated ubiquitination and subsequent degradation [334]. Only when cells are ready to enter anaphase (when all the chromosomes have attached to the spindle, creating tension on the spindle that satisfies the spindle assembly checkpoint [335]), Pds1 becomes dephosphorylated and is then promptly ubiquitinated by the APC. Subsequently, Pds1 degradation results in activation of Esp1, which cleaves cohesins to allow chromosome separation to take place. Furthermore, phosphorylation of Pds1 on a different set of Cdk1 sites is required to localize Esp1 to the nucleus, which may allow rapid activation of Esp1 once Pds1 becomes degraded [336]. As we will discuss in more detail in section 'Cdk1 and exit from mitosis', Cdc14-mediated dephosphorylation of the various Cdk1 sites of Pds1 creates a feedback loop that contributes to the switch-like behavior of anaphase onset, thus promoting synchronization of chromosome dissolution and separation by the spindle [334].

When cells enter M phase, the chromosomes condense to facilitate their segregation during anaphase. Chromosome condensation is mediated by the Smc2-Smc4 complex, which is structurally similar to the cohesin complex. Chromosome condensation is induced by CDK activity in vertebrates [337], in *Xenopus *egg extracts [338], and in *S. pombe *by phosphorylation of T19 on Cut3 (*S. pombe *Smc4). It is currently unknown whether Cdk1 is involved in stimulating condensin in *S. cerevisiae*, but it seems likely because Cdk1-induced chromosome condensation is evolutionarily conserved between *Xenopus *and *S. pombe*. An indication for an involvement of Cdk1 in regulation of the condensin complex comes from a recent study that followed decondensation of rDNA upon exit from mitosis [339]. In *S. cerevisiae*, rDNA condenses into a compact structure during M phase and this requires the binding of condensin [340-342]. When cells exit from mitosis (during which time Cdk1 becomes inactivated due to destruction of cyclins and expression of Sic1) the condensin component Brn1 is released from the rDNA, leading to rDNA decondensation [339]. Interestingly, the release of Brn1 from rDNA is inhibited by Cdk1, because when Cdk1 is artificially inactivated in anaphase-arrested cells, Brn1 is prematurely released from the rDNA; conversely, artificially sustaining Cdk1 activity during telophase results in delayed release of Brn1 [339]. Therefore, Cdk1 may either promote the association of condensin to rDNA or it inhibit its release; however, it is unclear what the relevant target of Cdk1 in this process is.

#### Regulation of spindle pole bodies

A crucial step in chromosome separation is assembly and alignment of the mitotic spindle, which partitions sister chromosomes to opposite poles. The division axis of the cell coincides with the mother-bud axis in budding yeast and is defined before formation of the mitotic spindle. Alignment of the spindle along this division axis and spatial coordination of spindle position with the cleavage apparatus is crucial to ensure proper inheritance of nuclei during cell division [343]. Both the assembly and alignment of the mitotic spindle are regulated by the spindle pole body (SPB; the *S. cerevisiae *microtubule-organizing center, or MTOC), which is inserted in the nuclear envelope [344]. The SPB is a cylindrical organelle that appears to consist of three plaques when visualized using EM: an outer plaque that is exposed to the cytoplasm and associates with cytoplasmic (astral) microtubules; an inner plaque that is exposed to the nucleoplasm and which associates with nuclear microtubules that in a later stage form the mitotic spindle; and a central plaque that spans the nuclear membrane to connect the inner and outer plaques [344]. One side of the central plaque is associated with a region of the nuclear envelope termed the half-bridge [344], a structure that is important for SPB duplication. SPB duplication takes place in several steps: First, the half-bridge elongates and deposits so-called satellite material, which serves as a seed for development of a new SPB; the second step is expansion of the satellite into a duplication plaque, after which the half-bridge retracts; the third step is insertion of the duplication plaque into the nuclear envelope and subsequent assembly of the inner plaque [344]. Finally, the bridge that still connects the old and new SPBs is severed, after which the SPBs move to opposite sides of the nuclear envelope. While it is beyond the scope of this review to discuss the structure and function of SPBs in further detail, we will highlight two Cdk1-controlled aspects of SPBs, i.e. SPB duplication and separation. An involvement for Cdk1 in duplication of spindle pole bodies was apparent from the analysis of the Hartwell *cdc *collection using electron microscopy [345], but it was not until recently that a key target of Cdk1 in this process, Spc42, was identified [346]. Spc42 is a protein that is essential for SPB duplication and which is thought to self-assemble to form a plaque [347,348]. It is present throughout the cell cycle and is phosphorylated during late G1 in a manner dependent on Cdk1 [347]. In addition to Cdk1, Mps1 is another kinase involved in SPB duplication [349], and Mps1 directly phosphorylates Spc42 [344]. Cdk1 directly phosphorylates both Spc42 and Mps1 [346]; Cdk1-mediated phosphorylation of Spc42 on S4 and T6 stimulates its insertion into the SPB, while Cdk1-mediated phosphorylation of Mps1 on T29 increases Mps1 stability. While an *spc42 *mutant in which both Cdk1 phosphorylation sites have been mutated to alanine can still duplicate SPBs, additional mutation of the Cdk1 site in Mps1 leads to poor viability of haploid cells and lethality of diploid cells [346]. In addition to phosphorylating Spc42 and Mps1, Cln-Cdk1 also stimulates the expression of SPB components by regulating SBF and MBF (see section 'Cdk1 and transcriptional programs'), thus contributing to SPB duplication. Notably, in a later stage of the cell cycle, mitotic Cdk1 (associated with either of Clb1,2,3,4) prevents re-duplication of the SPBs [350,351], which is important to prevent formation of a multipolar spindle due to the presence of more than two SPBs, which could result in missegregation of chromosomes and genomic instability. The exact molecular mechanism and the Cdk1 targets that participate in preventing re-duplication of SPBs remain unknown.

In addition to Spc42, the SPB component Spc110 also undergoes cell cycle-dependent phosphorylation, and similar to Spc42 this is mediated by both Mps1 and Cdk1 [352-354]. In particular, Clb-Cdk1 phosphorylates Spc110 on S36 and S91, and alanine substitutions of these sites cause mild spindle integrity problems, which lead to a spindle checkpoint-mediated mitotic delay [354]. The exact function of Spc110 phosphorylation by Mps1 and Cdk1 is not clear, but it may modulate the interaction between the microtubule-nucleating Tub4p complex and the SPB [353].

After duplication of the SPB, separation of the old and new SPBs in late S phase is crucial for successful assembly of the mitotic spindle and this is triggered by severing the bridge that connects the sister SPBs. After separation, the SPBs position themselves on the nuclear membrane such that they face each other, being separated by interconnecting microtubules to form what is generally referred to as a short spindle [355]. Separation of SPBs requires the kinesins Cin8 and Kip1 [356,357]; any of the cyclins Clb1,2,3,4 [358]; and dephosphorylation of Y19 of Cdk1 (phosphorylation of this residue by Swe1 inhibits Cdk1 activity) by Mih1 [359]. It was recently shown that dephosphorylation of Y19 of Cdk1 results in stabilization of Cin8, Kip1, and the spindle midzone component Ase1, which are thought to drive separation of SPBs by generating force, possibly by bundling microtubules [360]. Stabilization of these proteins is due to inhibition of the APC, which in absence of Cdk1 activity targets Cin8, Kip1 and Ase1 for destruction, and Cdk1 directly phosphorylates several APC components and inhibits the activity of the APC [360] (also see section 'Cdk1 and exit from mitosis'). Only when the balance between Swe1-mediated phosphorylation and Mih1-mediated dephosphorylation of Y19 on Cdk1 shifts towards a dephosphorylated state can Cdk1 phosphorylate and inhibit the APC, stabilizing Cin8, Kip1 and Ase1 and thereby driving SPB separation [361].

#### Attachment of chromosomes to the mitotic spindle

While the new SPB is still maturing, the nuclear microtubules emanating from the old SPB start capturing kinetochores. Kinetochores are large protein complexes that are formed on chromosome regions known as centromeres, DNA sequences of approximately 130 bp that contain the histone variant Cse4 (CENP-A in metazoans) [362-365]. Several protein complexes assemble onto the centromere, including (but not limited to) the Cbf3 complex, which directly binds to centromere DNA; the Ndc80 complex; the MIND complex; and the COMA complex [364]. While these complexes are involved in capture of microtubules, the attachment of microtubules to kinetochores is thought to be stabilized by the Dam1 complex (also known as the DASH complex) [364,366]. The chromosomal passenger complex consisting of the kinase Ipl1 (Aurora kinase) in complex with Sli15, Bir1 and Nbl1 phosphorylates Dam1 to facilitate the turnover of kinetochore-microtubule attachment until bi-orientation (binding of kinetochores to microtubules with opposite orientation) generates tension on kinetochores [367-369]. In addition to Dam1 phosphorylation by Ipl1, Cdk1 phosphorylates Ask1, another component of the Dam1 complex, on S216 and S250 during the S, G2 and M phases of the cell cycle [370]. Alanine substitution of these sites had little effect on cell viability when they were introduced into otherwise wild-type Ask1; however, when S216A and S250A substitutions were introduced into Ask1-3 (which is encoded by the temperature-sensitive *ask1-3 *allele), the result was exacerbated temperature-sensitivity [370]. In addition, the *ask1-3 *allele genetically interacted with hypomorphic *cdk1 *alleles, indicating that Cdk1 may function in attachment of microtubules to kinetochores [370]. While experimental evidence is lacking, Cdk1 may also affect this process by controlling the stability of Mps1 [346], which has recently been shown to be involved in kinetochore attachment [371].

#### Spindle positioning

Another important step in assembly of the mitotic spindle is spindle positioning, which involves alignment along the mother-daughter axis of division and placement at the bud neck [343,372-375]. Spindle positioning requires both the cytoplasmic microtubules that originate from SPBs as well as actin cables [376-378]. The initial alignment of the spindle requires asymmetric loading of Kar9 [233,379,380]; Kar9 localizes only to the SPB that is destined for the daughter cell, but not the mother-bound SPB. Loading of Kar9 onto the SPB appears to be regulated by microtubule-associated proteins (MAPs) Stu2 and Bim1. The Kar9-Bim1 complex is transported by kinesin from the minus ends of the cytoplasmic microtubules that emanate from the SPB to the tips of the microtubule plus ends located at the prospective daughter cell spindle pole [233]. Upon arrival at the plus ends of the microtubules Kar9 interacts with the actin-associated myosin Myo2, which then pulls Kar9 and the associated microtubule into the bud along actin cables that are polarized towards the bud (see section 'Cdk1 and cell morphogenesis'). After arrival at the bud, the microtubules are thought to be captured and linked to the bud cortex via Bud6 [381]. During anaphase, the final positioning of the spindle along the cell polarity axis is facilitated by the dynein-dynactin motor complex (targeted towards microtubule minus-ends), which pulls microtubules that are attached to the daughter-bound SPB through the bud neck [382-384]. The dynein-dynactin complex is recruited to the SPB by Bik1, which interacts with kinesin to promote transport of the dynein-dynactin complex to microtubule plus-ends [375]. Furthermore, like Kar9, the dynein-dynactin complex is also asymmetrically localized to the daughter-bound SPB, and the asymmetric localization of both Kar9 and dynein-dynactin contributes to correct positioning of the spindle. Importantly, the asymmetric loading of both Kar9 and dynein-dynactin is controlled by Cdk1, although the exact mechanism of Kar9 localization is still being debated [233,379,385]. Asymmetric loading of Kar9 was initially reported to be dependent upon its phosphorylation by Clb3,4-Cdk1 [233]. Another report doubted that Clb4 had an important role and suggested that it is Clb5-Cdk1 that mediates Kar9 localization instead [385]. More recent data indicate that both Clb4-Cdk1 and Clb5-Cdk1 complexes target different residues on Kar9; Clb5-Cdk1 may phosphorylate S496 while Clb4-Cdk1 may phosphorylate S197 [386]. The function of S496 phosphorylation may be to localize Kar9 to the SPB, while S197 phosphorylation might release Kar9 from Stu2, thus liberating it from the SPB [386]. Stu2 itself may also be a Cdk1 target, although the functional relevance of this phosphorylation is currently unclear [126,387]. While Cdk1-mediated phosphorylation of Kar9 is crucial for its asymmetric loading, the nature of the molecular determinants that mediate asymmetry remains unknown. It has been speculated that this may involve a daughter SPB-specific protein that binds phosphorylated Kar9 [380,386]. Alternatively, Cdk1 could have differential activities at the two SPBs, because Cdk1 is known to localize to SPBs and the localization and/or activity of cyclins Clb3 and Clb4 appear to be asymmetric as well [233,379], however the molecular basis for asymmetric Cdk1 activity is poorly understood. It is clear that the exact mechanism of asymmetric localization of Kar9 and the different Clb-Cdk1 complexes still remains to be established, and regarding its complexity and importance to the cell it likely involves the input from additional signaling pathways. Given that many processes that are controlled by the cell cycle involve feedback signaling, it would not be surprising if Kar9 affected Cdk1 activity to synchronize positioning of the spindle with cell cycle progression. It will be interesting to see how future studies will impact our current understanding of these processes.

Compared to Kar9, the asymmetric localization of the dynein complex occurs later in the cell cycle and depends on the mitotic cyclins Clb1,2 rather than Clb3,4 [388]. The activity of Clb1,2-Cdk1 on the dynein complex ensures unidirectional movement of the nucleus into the bud neck [389]. Cdk1 becomes inactivated during anaphase when Clb1,2 are destroyed and the phosphatase Cdc14 dephosphorylates Clb-Cdk1 targets, and this is thought to result in symmetric localization of the dynein complex to both SPBs, leading to movement of the two SPBs away from each other and elongation of the spindle [388,389]. The relevant Cdk1 target that mediates asymmetric localization remains unknown. Cnm67, a protein associated with the SPB, is required for the asymmetric localization of both the dynein complex as well as Clb2-Cdk1, and although it is phosphorylated on multiple sites by Clb2-Cdk1 *in vivo*, these phosphorylations are not required for dynein localization [388].

#### Spindle elongation

When all chromosomes have properly bi-oriented to create tension on the spindle and when the spindle is properly positioned, anaphase is triggered by Esp1/separase-mediated cleavage of the cohesin complexes, leading to spindle elongation. During this stage, the mitotic spindle is thought to be stabilized by Fin1, a self-associating coiled-coil protein that can form filaments between SPBs [390,391]. Fin1 is phosphorylated by Clb5-Cdk1 from S phase through metaphase [391,392], which inhibits the association of Fin1 with the spindle until Fin1 is dephosphorylated in anaphase due to degradation of Clb5 and activation of the phosphatase Cdc14 [392]. Fin1 dephosphorylation targets it to the poles and microtubules of the elongating spindle, where it contributes to spindle integrity and contributes to efficient chromosome segregation [392]. Fin1 is destroyed by the APC once cells have completed mitosis and started to disassemble the spindle [392].

Cdk1 also contributes to mitotic spindle stabilization and elongation by phosphorylating several components of the chromosomal passenger complex, which consists of Ipl1, Bir1, Sli15 and Nbl1, and which initially localizes to kinetochores to regulate their bi-orientation, but which relocalizes to the mitotic spindle during anaphase to control spindle stabilization and elongation. Cdk1 phosphorylates the passenger complex component Bir1 [393], resulting in recruitment of Ndc10, an inner kinetochore protein that binds to the centromere [394] but which relocalizes to the spindle midzone (the part of the mitotic spindle that constitutes interpolar microtubules that interdigitate between the two spindle poles to form an antiparallel microtubule array) in anaphase to promote spindle elongation [395]. Mutating the Cdk1 phosphorylation sites in Bir1 results in loss of Ndc10 from the anaphase spindle, increased chromosome loss and a defect in spindle elongation [393]. Furthermore, during metaphase Cdk1 phosphorylates Sli15 (inner centromere-like protein, or INCENP) within its microtubule-binding domain, which prevents its relocalization to the spindle. However, during anaphase the phosphatase Cdc14 dephosphorylates Sli15, resulting in relocalization of Sli15-Ipl1 to the spindle where it contributes to spindle stabilization [396]. Finally, another key Cdk1 target in organization of the mitotic spindle is Ase1, a microtubule bundling factor and a core component of the spindle midbody [397] that may also be involved in SPB separation. Cdk1 phosphorylates and inhibits Ase1 during metaphase, while during early anaphase dephosphorylation of Ase1 by Cdc14 promotes assembly of the spindle midzone [398,399]; midzone assembly is an important step in spindle elongation.

In conclusion, Cdk1 affects the assembly of the mitotic spindle in multiple ways: by controlling SPB duplication and separation, by positioning the spindle, by modulating kinetochore biorientation, and by promoting the assembly of the spindle midzone as well as stabilization and elongation of the mitotic spindle.

### Cdk1 and exit from mitosis

**Figure 6:**
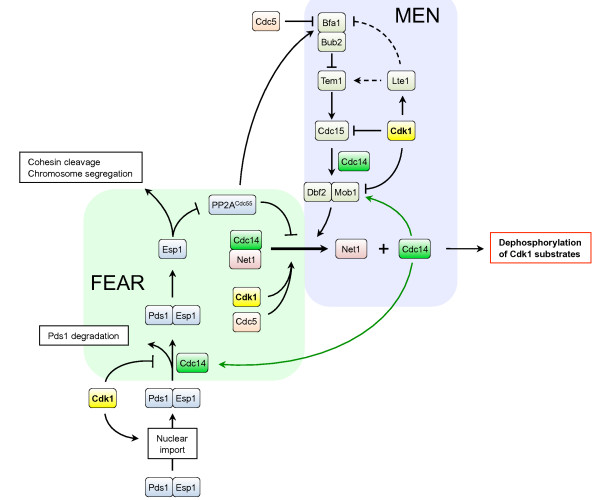
**The interplay between Cdk1 and mitotic exit**. Phosphorylation of Pds1 by Cdk1 results in nuclear import of the inactive Pds1-Esp1 complex, while phosphorylation of Pds1 on other Cdk1 sites protects it from degradation until cells are ready to initiate anaphase. Activation of the FEAR pathway and anaphase onset are encouraged by dephosphorylation of Cdk1 sites on Pds1 by the phosphatase Cdc14, which leads to degradation of Pds1 by the APC. Liberated from its inhibitor, Esp1 can now cleave cohesins and inhibit the phosphatase PP2A^Cdc55^. Downregulation of PP2A^Cdc55 ^shifts the balance from unphosphorylated Net1 to phosphorylated Net1, which is mediated by both Cdc5 as well as Cdk1, and results in dissociation of Cdc14 from Net1 and its release from the nucleolus. The release of a small amount of Cdc14 creates a positive feedback loop (green arrow) in which Cdc14 further dephosphorylates and thereby destabilizes Pds1, thus releasing more Cdc14. Downregulation of PP2A^Cdc55 ^also leads to a shift in the balance of unphosphorylated, active Bfa1-Bub2 to phosphorylated, inactive Bfa1-Bub2 (mediated by Cdc5), and downregulation of the GAP activity of Bub2 permits activation of the small GTPase Tem1. Lte1 may not directly activate Tem1, but rather indirectly through inhibiting Bfa1 by regulating its localization (dashed lines). Activation of Tem1 triggers the MEN, which provides the sustained Cdc14 activity that is necessary to exit from mitosis. Full activation of the MEN also requires dephosphorylation of the Cdk1 sites on Cdc15 and Mob1 by Cdc14.

The final steps of mitosis encompass an ordered series of events referred to as mitotic exit, which mediates the inactivation of Cdk1 and the dephosphorylation of key Cdk1 targets to reset the cell cycle (Fig. 6, for recent reviews see [400-403]). It starts with the separation of sister chromatids during anaphase upon Esp1-mediated loss of chromosome cohesion and involves elongation of the mitotic spindle. Once chromosome segregation is complete, the cytokinetic furrow is formed at the future site of cell division, the spindle disassembles, and cell division is completed by cytokinesis and abscission. During the past decade, tremendous progression has been made towards unraveling the molecular mechanisms that mediate mitotic exit, although it should be emphasized that the picture is far from complete. Here we focus mostly on the function of Cdk1 in mitotic exit.

Anaphase is triggered by ubiquitination and thereby proteasomal degradation of Pds1 (securin) by the APC, relieving inhibition of Esp1/separase, which subsequently cleaves the cohesion complex that holds together the sister chromatids. Simultaneously, the APC targets mitotic cyclins for destruction, leading to downregulation of mitotic Cdk1 activity, and destruction of Clb2 is particularly important for mitotic exit [404-406]. Further inhibition of Cdk1 activity is mediated by expression of the Cdk1 inhibitor Sic1, which occurs at the M-G1 boundary [404,406,407], and a feedback loop involving Sic1 ensures that mitotic exit is irreversible by preventing re-synthesis of mitotic cyclins [408]. In addition, Cdc6 has been reported to have a similar function in inactivation of Cdk1 by directly binding and inhibiting Clb-Cdk1 complexes [316,317]. However, Cdc6 may modulate mitotic exit at least in part through a Cdk1-independent mechanism by affecting the activity of the APC [314,315], and in addition Cdc6 may be less important for mitotic exit [316] than previously reported [317]. Finally, the phosphatase Cdc14 reverses phosphorylation of Cdk1 targets to reset the cell cycle to a basic G1 state; the activity of Cdc14 is paramount to mitotic exit [402,403], and in absence of Cdc14 activity cells arrest before cytokinesis in a telophase-like state with long spindles and a divided nucleus [409,410].

Cdk1 induces mitotic exit - and thus its own inactivation - by affecting the activity of the APC. APC activity fluctuates throughout the cell cycle in response to differential association with the activating subunits Cdc20 and Cdh1 (Hct1): during mid-mitosis it associates with Cdc20, leading to the initiation of anaphase, whereas during late mitosis it associates with Cdh1, and the APC^Cdh1 ^complex stays active throughout the subsequent G1 [411]. APC^Cdc20 ^and APC^Cdh1 ^have different substrate specificity; e.g. APC^Cdc20 ^targets Pds1 and APC^Cdh1 ^targets Ase1, while both APC^Cdc20 ^and APC^Cdh1 ^are required for full degradation of Clb2 [405,406,412]. There is extensive interplay between Cdk1 and APC activity; APC^Cdh1 ^degrades mitotic cyclins to inhibit Cdk1 activity [360,413,414], but upon entry of cells into S phase Cln1,2-Cdk1 and Clb5-Cdk1 phosphorylate Cdh1, blocking its interaction with the APC and thus allowing mitotic cyclins to build up again later in the cell cycle [413-415]. The interaction between Cdh1 and the APC is further inhibited by Cdk1-mediated phosphorylation and stabilization of Acm1, which inhibits Cdh1 by acting as a pseudosubstrate inhibitor [416-418]. Then at the end of mitosis Cdc14 dephosphorylates Cdh1, allowing it to interact with the APC again to destroy mitotic cyclins, thus completing the cycle [414,419]. Cdk1 is also required for activation of APC^Cdc20 ^during mitosis [420,421], which initiates the metaphase to anaphase transition by degrading Pds1 [406,422]. Cdk1 activates APC^Cdc20 ^by phosphorylating three components of the APC, Cdc16, Cdc23 and Cdc27, resulting in binding of Cdc20 to the APC [421]. Activation of APC^Cdc20 ^results in degradation of Pds1, leading to activation of Esp1 and thereby dissolution of chromosome cohesion, but it also leads to activation of the so-called FEAR (Cdc fourteen early anaphase release) network which results in transient activation of the phosphatase Cdc14 [422-424] (Fig. 6). Activation of the FEAR network is followed by activation of the mitotic exit network (MEN), which promotes sustained Cdc14 activity [402].

During most of the cell cycle, Cdc14 is sequestered in the nucleolus by Net1 (also known as Cfi1) [425,426]. The FEAR pathway is triggered by Esp1/separase-induced downregulation of the phosphatase PP2A^Cdc55^, which is apparently independent of the proteolytic function of Esp1 [427,428]. PP2A^Cdc55 ^keeps Net1 in a hypophosphorylated state, which promotes the interaction between Net1 and Cdc14 [428,429]. When Pds1 becomes degraded in early anaphase, Esp1 downregulates PP2A^Cdc55^, resulting in a shift in the phosphorylation balance of Net1 to a hyperphosphorylated state due to the action of Clb1,2-Cdk1 and Cdc5 [430-432]. Phosphorylation of Net1 abrogates the interaction with Cdc14 [430-432], which is then released from the nucleolus into the nucleus and cytoplasm to dephosphorylate Cdk1 targets. The FEAR network also encompasses additional proteins, such as the Esp1-associated protein Slk19; Tof2, which bears homology to Net1 [433]; Fob1, a nucleolar protein that localizes to rDNA and which interacts with Net1; and Spo12. Slk19 is a Cdk1 target, but the relevance of this is not well understood [387]. Fob1 forms a complex with Net1 and Spo12, and phosphorylation of Spo12 by Cdk1 contributes to activation of the FEAR pathway [423,434,435].

The initial release of Cdc14 is not sufficient for completion of mitotic exit, because when Cdk1 activity starts to drop during anaphase, Net1 could become hypophosphorylated again, which would then result in premature return of Cdc14 to the nucleolus before mitotic exit has been completed [423,428]. To circumvent this problem, cells activate the MEN to ensure sustained Cdc14 activity during late anaphase. The MEN pathway integrates information from the mitotic spindle with cell cycle progression [436,437]. A central component of the MEN is a small Ras-like GTPase named Tem1, which localizes to the daughter-bound SPB [436-438]. The MEN is thought to be activated when the daughter-bound SPB moves into the bud, which is the compartment where Lte1 is located, a protein with similarity to GTP-exchange factors that localizes only to the bud and which may induce the activity of Tem1 [436,437,439]. Lte1 may not directly activate Tem1, but rather indirectly activates Tem1 by inhibiting Bfa1, which is an inhibitor of Tem1 [440] (also see below); the asymmetric localization of Lte1 to the bud cortex is mediated by Cdk1 and Cla4 [441-443]. Active Tem1 then activates a signaling cascade by interacting with the kinase Cdc15, which in turn activates the Mob1-Dbf2 kinase complex [444-448]. Exactly how Mob1-Dbf2 then promotes Cdc14 release from the nucleolus is not well understood [403], but it involves direct phosphorylation of Cdc14 on serine and threonine residues adjacent to a nuclear localization signal (NLS), thereby abrogating its NLS activity resulting in nuclear exclusion [449]. This then promotes mitotic exit.

It is important that the MEN pathway is not activated before chromosome separation is complete, as this could result in missegregation of chromosomes. Premature activation of the MEN pathway is prevented by multiple means. Tem1 is kept inactive at the SPB by the GAP Bub2-Bfa1. Like Net1, Bfa1 is kept in a hypophosphorylated state by PP2A^Cdc55 ^during metaphase, but when PP2A^Cdc55 ^is downregulated by Esp1 during early anaphase, the balance shifts towards hyperphosphorylated Bfa1, which is mediated by Cdc5. Phosphorylation of Bfa1 inhibits its activity and therefore results in activation of Tem1 and hence mitotic exit. Bfa1 is also regulated by the spindle positioning checkpoint (SPOC), which delays mitotic exit when the anaphase spindle fails to extend toward the mother-daughter axis [450]. When the mitotic spindle is misaligned, the kinase Kin4 phosphorylates Bfa1, which prevents phosphorylation and inhibition of Bfa1 by Cdc5 [451-453]. Because Cdc5 cannot phosphorylate and inhibit Bfa1, Bfa1 continues to block Tem1 activity, thereby preventing mitotic exit. However, during an unperturbed cell cycle - when the spindle is properly aligned - Kin4 localizes to the mother SPB, while Bfa1 localizes to the daughter SPB; as a result of this differential localization, Kin4 cannot phosphorylate Bfa1, which then becomes phosphorylated by Cdc5 instead, leading to inhibition of Bfa1, activation of Tem1, and mitotic exit [453]. Interestingly, the asymmetric localization of Bfa1 was recently reported to be promoted by Lte1 [440]. Thus, Lte1 may activate Tem1 indirectly by inhibiting Bfa1 rather than directly through its GEF domain [440].

Full activation of the MEN pathway requires Cdc14-mediated dephosphorylation of Cdc15 and Mob1, both of which are targets of Cdk1 [454,455]. Phosphorylation of Cdc15 and Mob1 is inhibitory, and their dephosphorylation by Cdc14 may contribute to fine-tuning of MEN activity, but it may also ensure a right order of events, such that MEN does not take place before activation of the FEAR network (which releases Cdc14 which can then dephosphorylate Cdc15). Ultimately, when cells have successfully exited from mitosis, Cdc14 is downregulated by its return to the nucleolus, which is mediated by degradation of Cdc5 by the APC [456], which results in a shift in the phosphorylation balance of Net1 and Bfa1 to a hypophosphorylated state.

### Cdk1 in maintenance of genome stability

**Figure 7:**
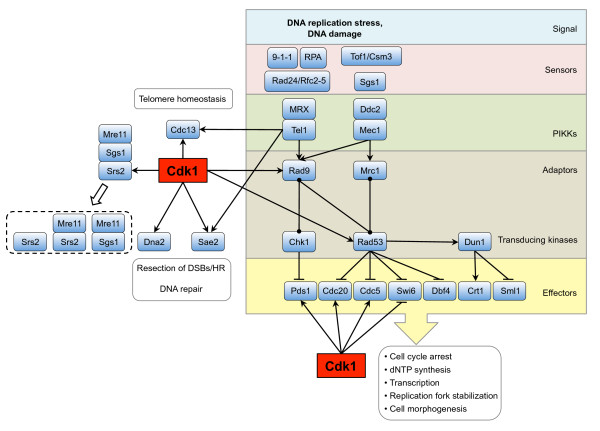
**Cdk1 modulates the activity of several DNA damage checkpoint proteins**. DNA damage and replication stress are sensed by a number of proteins that activate the PIKKs Mec1 and Tel1. These kinases activate a signal transduction pathway consisting of the adaptor proteins Mrc1 and Rad9 and the kinases Rad53, Dun1 and Chk1. Cdk1 may phosphorylate Rad9 to boost the signaling cascade. Cdk1 also phosphorylates Rad53, which may prevent checkpoint adaptation, but which may also affect processes involved in cell morphogenesis. Together, Mec1, Tel1, Rad53, Dun1 and Chk1 phosphorylate a number of effector proteins (only a subset of effectors is shown in this figure) that mediate the DNA damage response. Several of these effectors are also targeted by Cdk1, although the consequence of simultaneous phosphorylation by Rad53 and Cdk1 is unclear. Mec1/Tel1 and Cdk1 directly phosphorylate Sae2 to stimulate its nuclease activity, which is important for resection of DSBs, thereby channeling DSBs into the HR pathway. Full resection of DSBs also requires the activity of Dna2 and Sgs1. Phosphorylation of Dna2 by Cdk1 increases its nuclear import, while Cdk1 may affect Sgs1 by phosphorylating Srs2, which leads to formation of subcomplexes consisting of Srs2, Srs2-Mre11 and Sgs1-Mre11. Mec1/Tel1 and Cdk1 also directly phosphorylate Cdc13, resulting in recruitment of telomerase and telomere elongation. See text for details.

Proper regulation of the cell cycle is required to transmit a complete and intact copy of the genome from one generation to the next. Cdk1 commands the cell cycle and as has become clear in previous sections, it is involved in many aspects of DNA metabolism. It is therefore not surprising that defects in regulation of Cdk1 have been found to result in genome instability. The role of Cdk1 on maintenance of genome stability can be broken down into two main functions: The first is preventing DNA damage and genome instability by regulating the processes involved in replication and segregation of DNA; and the second is the regulation of DNA repair processes after DNA damage has occurred. These two functions of Cdk1 are not necessarily mutually exclusive; e.g. premature initiation of DNA replication due to aberrant Cdk1 activity can produce DNA lesions that may subsequently not be accurately repaired because their repair also requires proper Cdk1 activity. As discussed in previous sections, Cdk1 affects a number of processes that could give rise to genome instability when improperly regulated. In addition, Cdk1 has been found to directly control a number of targets involved in the DNA damage response (Fig. 7, also see previous sections). In this section we will discuss the role of Cdk1 in maintenance of genome stability.

Different forms of genome instability exist [457] and they can roughly be divided into two classes: (i) changes in chromosome number (often referred to as chromosomal instability, or CIN), and (ii) alterations at the DNA sequence level (which we will refer to as genomic instability, or GIN). CIN can be caused by failures in either mitotic chromosome transmission or the mitotic spindle checkpoint, resulting in aneuploidy and loss of heterozygosity [335], while GIN can be caused by problems during DNA replication and repair, resulting in the accumulation of mutations and genome rearrangements. Some forms of GIN involve changes at the nucleotide level [458] (e.g. single base changes, addition or loss of one or several nucleotides) which can be the result of defects in DNA repair processes such as mismatch repair (MMR), base excision repair (BER), nucleotide excision repair (NER), or by error-prone translesion synthesis. GIN at a larger scale involves loss or amplification of parts of chromosomes, often referred to as gross chromosomal rearrangements (GCRs), such as translocations, duplications, inversions or deletions [459].

#### Chromosome instability

CIN can be caused by numerous problems during the mitotic cell cycle. For instance, defects in chromosome cohesion reduce the fidelity of chromosome segregation [460-462]. Furthermore, cells with multipolar spindles (due to an aberrant number of SPBs) as well as merotelic attachments (i.e. the simultaneous attachment of one kinetochore to microtubules emanating from both spindle poles rather than a single pole) are likely to missegregate their chromosomes [463-465]. Finally, aneuploidy can also result from chromosome missegregation produced by defects in the mitotic checkpoint, which ensures attachment of all chromosomes to the mitotic spindle; in mutants with a defective mitotic checkpoint anaphase initiates before all chromosomes have established proper spindle attachments [335,466].

Cdk1 is known to affect chromosome transmission fidelity, and both aberrantly increased Cdk1 activity, e.g. in *sic1Δ *mutants, as well as reduced Cdk1 activity, e.g. in *cdk1 *point-mutants, leads to increased rates of chromosome loss [73,467-469], indicating that Cdk1 activity must be carefully balanced throughout the cell cycle in order to prevent CIN. However, the role of Cdk1 in this process is not well defined. Cdk1 controls many processes that could lead to CIN if improperly regulated. For instance, Cdk1 activity is required for chromosome cohesion, and it prevents premature loss of cohesion by phosphorylating and thereby inhibiting Pds1 degradation [334]. Cdk1 also controls duplication of SPBs early in the cell cycle while preventing SPB re-duplication later in the cell cycle, and failure to either duplicate SPBs or to prevent re-duplication may result in monopolar or multipolar spindles and missegregation of chromosomes. Furthermore, the attachment of microtubules to the SPBs as well as the kinetochores may be controlled by Cdk1, and Cdk1 also regulates the assembly, positioning and elongation of the mitotic spindle; improper attachment of chromosomes to the spindle or aberrant spindle behavior can lead to CIN [335]. Finally, Cdk1 may be important for the mitotic checkpoint that monitors spindle assembly [328], and defects in this checkpoint are well-known to contribute to CIN [335]. Although Cdk1 has not always been directly linked to CIN in these processes, it is clear that its activity must be carefully regulated to prevent CIN.

#### Genome instability

In addition to affecting CIN, Cdk1 is involved in a number of cellular processes that could lead to GIN if not properly regulated. As we will discuss below, Cdk1 promotes DNA replication but inhibits re-replication, it may be involved in activation of S phase checkpoints, and it stimulates DNA repair. Here we will focus on one form of GIN that we and others have found to be affected by Cdk1, i.e. GCRs.

The majority of GCRs are thought to stem from problems during DNA replication [459]. When a DNA replication fork stalls upon encountering a lesion in the DNA (e.g. UV-induced cross-linked nucleotides, bulky DNA adducts, etc.) and the problem is not rectified, the fork is at risk of collapse, potentially leading to DNA double strand breaks (DSBs). Stalled replication forks and DSBs activate the DNA replication checkpoint and the DNA damage checkpoint. These checkpoints are defined as the pathways that promote cell cycle delay or arrest in response to DNA replication stress or DNA damage, respectively [470]. The central dogma for cell cycle checkpoints is often presented as: DNA damage signals → damage sensors → signal transducers → effectors [471]. Exactly how DNA replication stress and DNA damage are sensed is not clear, but it involves the presence of single-stranded DNA (ssDNA) and a number of proteins including (but not limited to) the ssDNA binding complex RPA; the Rad24-RFC complex which loads the Ddc1-Rad17-Mec3 clamp (also referred to as the 9-1-1-complex), which may also serve as a sensor; the helicase Sgs1; Tof1-Csm3; and the Mre11-Rad50-Xrs2 (MRX) complex [471] (Fig. 7). The next step in checkpoint activation is the recruitment of the phosphoinositide 3 kinase-related kinases (PIKKs) Mec1 (similar to metazoan ATR) and Tel1 (similar to ATM). Mec1 is recruited to stalled forks and DSBs by Ddc2, while Tel1 may be recruited to DSBs by interacting directly with the MRX complex [472]. Activation of Mec1 and Tel1 results in recruitment and phosphorylation of the adaptor proteins Mrc1 and Rad9, which in turn recruit and activate the kinases Chk1 and Rad53 (similar to mammalian Chk2) [473]. Rad53 appears to be the main player, especially in terms of stabilization of replication forks during DNA replication stress, although Chk1 has functions in stabilizing replication forks in the absence of Rad53 [474]. Checkpoint activation in *S. pombe *and higher eukaryotes results in inhibition of Cdk1 by stabilizing the phosphatase Cdc25, thereby shifting the balance from unphosphorylated to Y19-phosphorylated, inactive Cdk1. Furthermore, higher eukaryotes also activate p53 to induce the expression of CKIs such as p21 to further inhibit CDK activity. While DNA damage in an early stage of the cell cycle may delay entry into S phase by inhibiting Cdk1 (through Rad53-mediated phosphorylation and thereby inhibition of the transcription factor Swi6, preventing expression of *CLN1 *and *CLN2 *[475,476], *S. cerevisiae *cells typically arrest the cell cycle with high Cdk1 activity, and inhibitory phosphorylation of Y19 of Cdk1 is not required for efficient cell cycle arrest [477,478]. Instead, checkpoint activation induces cell cycle arrest by directly targeting the processes that are required for cell cycle progression; for example Rad53 inhibits firing of late origins of replication [479,480] at a stage after pre-RC formation but before pre-IC formation, and it has been shown to phosphorylate the Dbf4-Cdc7 kinase complex (DDK, which is involved in pre-IC formation, see section 'Cdk1 and DNA replication'), which may inhibit DDK activity and remove it from chromatin [481-484]. In addition to inhibiting late origin firing, activation of the checkpoint is thought to block cell cycle progression by inhibiting chromosome segregation through Chk1-mediated phosphorylation and thereby stabilization of Pds1, thus preventing activation of Esp1 and loss of cohesion [485-490]. Mec1 and Rad53 further prevent mitotic progression by inhibiting the APC component Cdc20 [491,492], and Mec1 blocks spindle elongation by inhibiting the expression of Cin8 and Stu2 [493]. Finally, the checkpoint may enforce cell cycle arrest by inhibiting Cdc5 to prevent mitotic exit [489]. Besides checkpoint activation to inhibit cell cycle progression, the DNA damage response includes upregulation of ribonucleotide reductase to produce more dNTPs by phosphorylation and degradation of the RNR inhibitor Sml1 by Dun1 [494-496]; induction of transcriptional programs by Dun1-mediated phosphorylation and thereby inhibition of the transcriptional repressor Crt1 [497]; stabilization of DNA replication forks [498] and replication fork restart [499], recruitment of DNA repair factors [471,500], coordination of cell morphogenesis through timely degradation of Swe1 [81,501], and inhibition of nuclear migration [502]. The importance of an intact DNA damage response in maintenance of genome stability is underscored by the finding that checkpoint defective mutants have high rates of GCRs [503-505], and as we will discuss below, Cdk1 modulates checkpoint activation as well as DNA repair pathways.

#### Cdk1 in checkpoint activation and DNA repair

Several studies have shown that Cdk1 activity must be carefully regulated in order to prevent DNA damage. For example, failure of Cdk1 to prevent re-replication induces DNA damage [506,507]. Increased Cdk1 activity (either by deleting *SIC1 *or by overexpression of a stabilized form of Cln2), which induces premature entry into S phase, leads to DSBs and the formation of GCRs [325,468,508], and overexpression of either *CLN1 *or *CLN2 *requires a functional checkpoint for viability [509]. Conversely, reduced Cdk1 activity (by depleting the S phase cyclins Clb5 and Clb6) also triggers a checkpoint response [510,511], indicating the formation of DNA damage. Furthermore, *clnΔ cln2Δ *double mutants require functional Rad27 (the *S. cerevisiae *version of the flap endonuclease Fen1 that processes Okazaki fragments; cells lacking Rad27 have high levels of DSBs and high GCR rates [512]) for viability, as do mutants expressing hypomorphic *cdk1 *alleles [325,513]. These findings indicate that Cdk1 is required for the cellular response to DSBs that occur due to loss of Rad27 activity. Finally, reduced Cdk1 activity (by loss of expression of Clb5,6 or expression of hypomorphic *cdk1 *alleles) leads to sensitivity to various forms of DNA damage [325,510,514], providing additional evidence that Cdk1 is involved in the DNA damage response. Together, these studies show that the activity of Cdk1 must be tightly regulated, because either too much or too little Cdk1 activity leads to DNA damage and genome instability, and these studies also suggest a potential involvement of Cdk1 in the DNA damage response. Indeed, Cdk1 has been shown to be required for DSB-induced checkpoint activation and for homologous recombination (HR) [515], and for recruitment of the HR protein Rad52 to DSBs [516]. *S. cerevisiae *cells preferentially repair DSBs through HR during S, G2 and M phase, when there is a template present to carry out HR. However, in G1, when there is no template present for HR, cells repair DSBs by non-homologous end-joining (NHEJ). While this differential cell cycle-dependent repair of DSBs was initially thought to be passive (i.e. because there is no template in G1, cells automatically channel DSBs into the NHEJ pathway), it was recently discovered that cells actively determine the pathway of DSB repair, and that this depends on Cdk1 activity [515]. When Cdk1 is inactive (in G1 phase), the default form of repair is NHEJ, however when Cdk1 is active (S-G2-M) the cell preferentially uses HR for DSB repair. The effect of Cdk1 appears to be two-fold; it actively promotes HR during S-G2-M [515], while simultaneously actively suppressing the recruitment of proteins involved in NHEJ [517]. While the mechanism of suppression of NHEJ by Cdk1 is unknown [517], the mechanism by which it stimulates HR is much better defined: it phosphorylates the nuclease Sae2, which induces it to resect DSBs to expose ssDNA [518], which is the first step of HR [519,520]. Furthermore, the exposed ssDNA is thought to promote checkpoint activation [515]. However, it should be noted that Sae2 only resects a relatively small amount of DNA, and efficient resection of DSBs requires the additional activity of Mre11-Rad50-Xrs2 complex, the nucleases Dna2 and Exo1, and the helicase Sgs1 [521-523]. Furthermore, an *sae2Δ *deletion mutant is not as sensitive to DNA damaging agents like MMS as hypomorphic *cdk1 *mutants, indicating that Cdk1 must have additional targets in the DNA damage response (our unpublished results). One such target may be Dna2, which is a very efficient *in vitro *substrate for Cdk1 [126], and it was recently shown that phosphorylation of Dna2 in its NLS by Cdk1 may target it to the nucleus [118]. Therefore, it is tempting to speculate that Cdk1 drives a concerted effort to resect DSBs by activating Sae2 and inducing nuclear import of Dna2.

It should be noted that although Cdk1 may be required for HR, we have recently demonstrated a genetic interaction between *CDK1 *and *MRE11 *(as well as other components of the MRX complex), and we also found that Mre11 and Cdk1 cooperate to prevent mitotic catastrophe after HU-induced DNA replication stress [325]. These results suggest that while Cdk1 may promote HR by stimulating Sae2, it is likely to have an additional function in a pathway parallel to HR, although the nature of this pathway is currently unknown.

In addition to phosphorylating Sae2, Cdk1 also targets the helicase Srs2 [524,525]. Srs2 is complexed to Mre11 and Sgs1 during unperturbed conditions, but treatment of cells with MMS leads to formation of Srs2-Mre11, Srs2 and Sgs1-Mre11 subcomplexes [525]. Although the physiological relevance of formation of these subcomplexes is not well defined, it depends on Srs2 phosphorylation by Cdk1, and mutation of these phosphorylation sites results in sensitivity to the DNA alkylating agent MMS [525]. In a more recent study, detailed analysis of Cdk1-mediated phosphorylation of Srs2 revealed that it inhibits Srs2 sumoylation while promoting the helicase function of Srs2 during HR [526]. How this relates to the MMS-induced formation of the Srs2-Mre11, Srs2 and Sgs1-Mre11 subcomplexes remains unknown.

Cdk1 has been reported to be required for checkpoint activation [515]. However, there are conflicting reports regarding the involvement of Cdk1 in checkpoint activation. One study showed that ionizing radiation and HO-induced DSBs require Cdk1 activity for full activation of Rad53 in G2/M phase-arrested cells while these treatments did not activate Rad53 in G1 phase [515], and activation of Rad53 by MMS treatment did not require Cdk1 during G2/M [515]. In support of this study, artificial activation of Rad53 by colocalization of upstream checkpoint sensors (but in absence of DNA damage) requires Cdk1-dependent phosphorylation of Rad9 in G2/M-phase arrested cells [527]. In contrast, other studies found that inhibition of Cdk1 does not block HU-induced activation of Rad53 [325,524]. One explanation for this apparent discrepancy might be a differential response of cells to various DNA damaging agents, i.e. DSBs induced by ionizing radiation or by HO breaks require Cdk1, while HU-induced DNA replication stress does not. Alternatively, cells may respond differentially to DSBs during different stages of the cell cycle, since DSBs that occur during G2/M phase lead to a moderate activation of the checkpoint, while DSBs that occur during S phase lead to much stronger checkpoint activation due to replication fork stalling [528]. Therefore, checkpoint activation by stalled replication forks may either not require Cdk1 activity, or the checkpoint response is so strong that it overrides the requirement for Cdk1. A third explanation might be redundancy in checkpoint activation, because it was recently shown that Cdk1 by itself is not sufficient to activate the checkpoint during S phase, but it also requires the activity of the Ddc1-Rad17-Mec3 complex [529].

Interestingly, Cdk1 was recently shown to directly phosphorylate Rad53 on S774 [530,531]. Phosphorylation of Rad53 by Cdk1 does not appear to have consequences for checkpoint activation [530,531], but may rather prevent checkpoint adaptation [530]; checkpoint adaptation is defined as the process in which a cell resumes the cell cycle even though DNA damage is still present [532]. Another study also did not find a function for Cdk1-mediated phosphorylation of Rad53-S774 in checkpoint activation [531]. Instead, it was shown that this phosphorylation may be important for Rad53's function in regulating cell morphogenesis [531], which we previously found to promote cell viability during DNA replication stress [81]. More detailed studies should clarify the exact functions of Cdk1 in the DNA damage response during the various stages of the cell cycle and upon different forms of DNA damage.

In addition to mediating HR-dependent DNA repair and modulating the DNA damage checkpoint, Cdk1 also controls at least three aspects of telomere homeostasis: (i) cell cycle-dependent telomere elongation, (ii) resection and degradation of telomeres in mutants lacking full telomerase activity, and (iii) HR-dependent telomere extension in post-senescent survivors that arise in telomerase-deficient cells.

Telomeres protect the chromosomes against degradation by DNA repair enzymes and checkpoint proteins that otherwise might recognize the chromosome ends as DNA double-strand breaks [533,534]. Telomeres are elongated by replication by telomerase, a ribonucleoprotein enzyme that synthesizes DNA by using its own RNA moiety as a template, thus overcoming the end-replication problem (i.e. loss of sequence and chromosome degradation as cells divide) [535,536]. Telomere protection and telomerase recruitment are mediated by Cdc13, a ssDNA binding protein that directly interacts with telomerase (Est1) [537-540]. Telomere elongation is cell cycle dependent, and recently it was shown that Cdk1-mediated phosphorylation of Cdc13 promotes its interaction with Est1, leading to telomere elongation [541,542]. In contrast, when Cdc13 is depleted from cells by growing temperature-sensitive *cdc13-1 *mutants at restrictive temperature, the telomeres are resected in manner dependent on Cdk1, resembling Cdk1-dependent resection of a DSB [515,543]. Therefore, the Cdk1-dependent resection of dysfunctional telomeres that form due to absence of Cdc13 may be an attempt to repair chromosome ends that are now recognized as DSBs. Furthermore, in telomerase-deficient mutants (which senesce due to erosion of telomeres), rare post-senescent survivors arise that utilize HR to elongate telomeres in a telomerase-independent fashion. Interestingly, Clb2-Cdk1 has been found to be required for formation of such HR-dependent post-senescent survivors [544,545], again resembling the Cdk1-dependent processing of a DSB by the HR pathway. However, it is currently unknown which enzyme is targeted by Cdk1 to induce resection of the chromosome ends, and although it is tempting to speculate that it might be Sae2, *SAE2 *has not been identified in screens for genes that affect telomere length [546,547], and in addition Ctp1 (a diverged ortholog of Sae2 in *S. pombe *[548]) does not appear to have a role in telomere homeostasis [548]. One last target of Cdk1 is worth mentioning in respect to processing of DSBs: Mer2, a Spo11 ancillary protein required for DSB formation during meiosis. Clb5-Cdk1 was already known to be required for formation of DSBs during meiosis [549], but recently Clb5-Cdk1 was shown to directly phosphorylate Mer2 on S30 and S271 during meiosis [550]. Phosphorylation of S30 may serves as a priming site for phosphorylation by DDK on S29 [551,552], and collectively these phosphorylations may promote the loading of Spo11 on meiotic recombination hotspots, possibly by interaction with Mei4, Rec114 and Xrs2 [550]. Therefore, Cdk1 is involved in processing of DSBs during the mitotic phase of the cell cycle as well as in formation and processing of DSBs during meiosis.

There exists overlap between targets of Cdk1 and the kinases that mediate the DNA damage response (see Fig. 7) (for recent reviews on targets of checkpoint kinases see [321,471,553]). For instance, Sae2 is phosphorylated by Mec1/Tel1 [554] and Cdk1 [518], and mutating either its Mec1/Tel1 sites or its Cdk1 phosphorylation site results in increased sensitivity to DNA damage, indicating a concerted response of checkpoint kinases and Cdk1 to DNA damage. Another example is Cdc13, which is phosphorylated on several sites by Mec1 and Tel1 as well as by Cdk1 to promote recruitment of telomerase in order to maintain telomere length [555]. Other proteins that are targeted both by Cdk1 and checkpoint kinases are Swi6, Cdc5, Cdc20 and Pds1. It is currently unclear why overlap between targets exists, and it remains unknown to what extent the combined checkpoint-mediated and the Cdk1-mediated phosphorylations affect the function of the protein to determine the final output of the DNA damage response. Presumably, the fact that Cdk1 and the checkpoint kinases converge on an overlapping set of targets helps coordinate the DNA damage response with the cell cycle.

An important function of the DNA damage response and DNA repair pathways is to suppress genome instability. One form of genome instability, GCRs, is suppressed by many checkpoint and DNA repair proteins [459,556]. Most GCRs are thought to arise from problems during DNA replication, which might be the result of lesions and replication blocks that are improperly processed. For instance, reactive oxygen species (ROS) can cause serious problems during DNA replication resulting in genome instability because they can induce many types of DNA damage, including single- and double-stranded DNA breaks, base and sugar modifications, and DNA-protein crosslinks [557,558], and mechanisms that protect the cell against the deleterious effects of ROS cooperate with various DNA repair pathways such as HR to suppress GCRs [559]. Another cause of GCRs is DNA replication itself, especially in mutants in which the fidelity of DNA replication is reduced [512,560], and GCRs also arise in mutants that are defective in assembly of newly replicated DNA into chromatin [561]. Furthermore, the activity of the S-phase checkpoint and various DNA repair pathways are essential for suppression of GCRs [503-505], as is proper regulation of the processes that control telomere formation and maintenance [562,563]. Finally, we and others have shown that Cdk1 is involved in formation of GCRs [325,468,508].

As discussed in previous sections, Cdk1 plays multiple roles in DNA replication; low Cdk1 activity in G1 promotes pre-RC formation, while Cdk1 activity during S phase results in origin firing and prevents re-replication. It is therefore not surprising that increased Cdk1 activity (due to depletion of Sic1 or Far1, or by overexpression of a stabilized form of Cln2) leads to increased GCR rates [325,468,508]. Presumably, this is due to premature entry into S phase, when either not enough pre-RCs have been assembled or pre-RC assembly is still incomplete [468], and consistent with this the addition of multiple origins can suppress the increased GCR rate of cells overexpressing Cln2 [508]. What is more surprising, however, is the finding that Cdk1 activity is not just necessary but in fact also required for formation of GCRs [325]. Reduced Cdk1 activity (by expression of hypomorphic *cdk1 *alleles) is able to suppress the very high GCR rates that are observed in mutants lacking proteins involved in DSB repair, such as Mre11, but also the flap endonuclease Rad27, the helicase Pif1 (which suppresses de novo telomere additions), and S phase checkpoints [325]. In contrast, hypomorphic *cdk1 *alleles do not suppress small-scale mutations that arise in *msh2Δ *mismatch repair mutants. This indicates that Cdk1 specifically prevents formation of rearrangement-prone forms of DNA damage, such as single-strand and double-stranded DNA breaks, or alternatively it processes these forms of damage once they have occurred. Exactly how Cdk1 is required for formation of GCRs is currently unknown, although it cannot be explained by simply a reduced speed of cell cycle progression (due to reduced Cdk1 activity), which could give cells more time to faithfully repair DNA damage to evade formation of a GCR [325]. Furthermore, the requirement for Cdk1 in formation of GCRs is not mediated by the Sae2-HR pathway, because deletion of Sae2 increases rather than suppresses GCRs [325]. It is more likely that Cdk1 promotes GCR-prone repair of damaged chromosomes, and that in absence of Cdk1 activity repair does not take place, resulting in loss of the broken chromosome and subsequent inviability due to loss of essential genetic information, leading to an apparent reduction in GCR rates. What then might be the mechanism for Cdk1 in formation of GCRs? One clue comes from a set of genes that, like *CDK1*, have been found to be required for formation of GCRs, and deletion of these genes also results in suppression of GCRs, similar to hypomorphic *cdk1 *alleles [564]. These genes, *BUB1*, *BUB2*, *BUB3*, *MAD2 *and *MAD3*, are involved in the mitotic spindle checkpoint and mitotic exit. As discussed in previous sections, Cdk1 may be involved in these processes, and one could speculate that the requirement for Cdk1 in formation of GCRs involves these gene products. Interestingly, we found genetic interactions between *CDK1 *and genes involved in these processes, suggesting they share common functions [325]. It is currently unknown how these genes are required for formation of GCRs, but it is of interest to note that treating cells with a low dose of nocodazole (which severely slowed the cell cycle due to activation of the mitotic spindle checkpoint), resulted in increased GCR rates [325], again suggesting that an intact mitotic spindle checkpoint is somehow required for formation of GCRs. One explanation for this observation could be that the activity of the mitotic spindle checkpoint ensures that cells spend a little more time in M phase, at least long enough for GCR-prone healing of any broken chromosomes to occur; in absence of the mitotic checkpoint M phase lasts shorter and cells with any broken chromosomes might now exit from mitosis before chromosome healing has taken place, which subsequently results in chromosome loss and inviability, leading to an apparent reduction in GCR rates. Whether Cdk1 indeed exerts its effect on formation of GCRs through its spindle assembly function remains to be determined.

In conclusion, Cdk1 affects many aspects of CIN and GIN. It has positive effects on genome stability by preventing mitotic catastrophe, however it negatively affects genome stability by promoting formation of GCRs. Cdk1 activity needs to be carefully regulated, because either too much or too little Cdk1 activity can affect genome integrity.

### Future directions and ramifications for cancer treatment

Many aspects of the cell cycle are directly controlled by Cdk1, and include regulation of cell polarity and morphology, DNA replication, chromosome segregation, and maintenance of genome stability. Many, if not all, facets of Cdk1 regulation involve positive and negative feedback loops, reflecting the need for tight control of the cell cycle. This is especially evident in regulation of processes that affect genome stability, because both an aberrant increase as well as a decrease in Cdk1 activity can lead to genome instability, with potentially disastrous consequences for the organism. While regulation of Cdk1 activity is relatively well understood, comparatively little is known about its downstream targets. As discussed in this review, approximately 75 Cdk1 targets have been described in *S. cerevisiae *(See additional Table 1), but regarding the enormous complexity of cell duplication, we expect many more to be identified. While the use of classic yeast genetics has been useful in the discovery of upstream regulators of Cdk1, such as cyclins and Cak1, downstream components are rarely identified in suppressor screens, probably because Cdk1 activity is required for several essential cellular processes throughout the cell cycle, and no single Cdk1 target can compensate for loss of Cdk1 activity during all these different steps. More advanced genetic screens may be required to unravel the complete genetic network of the cell cycle that involve *CDK1*, like e.g. synthetic genetic array (SGA) and synthetic dosage lethality (SDL) screens, which have been successful in identification of novel processes and targets controlled by the related CDK Pho85 [191,565-568]. Furthermore, in a recent study, which combined specific chemical inhibition of Cdk1 with quantitative mass spectrometry, 308 potential Cdk1 substrates were identified [17], many of which had previously been shown to be *bona fide *Cdk1 targets. The functional consequences of phosphorylation of the vast majority of these potential Cdk1 substrates still needs to be determined.

Complexity to Cdk1 signaling is added by the fact that multiple enzymes can recognize Cdk1 phosphorylation sites to further modify those proteins; e.g. the proline isomerase Ess1/Pin1 can be recruited to phosphorylated SP/TP sites (potentially phosphorylated by Cdk1) to isomerize the proline residue, and this has been shown to affect diverse cellular processes, including growth factor-induced signal transduction pathways, cell-cycle progression, cellular stress responses, neuronal function and immune responses [569]. Additionally, phosphorylation by Cdk1 can serve as a priming site for further phosphorylation by other kinases, such as the polo kinase Cdc5 [135]. Furthermore, there exists extensive cross-talk between Cdk1 and Pho85 [8]. Potential cross-talk between Cdk1 and the other CDKs (Ssn3, Kin28, Bur1 and Ctk1) remains largely unexplored, although cross-talk might be expected based on the fact that Cdk1 and the other CDKs all control various facets of transcription. Other aspects of Cdk1 signaling have remained obscure, e.g. Cdk1 has a kinase-independent role in regulation of transcription, but little more is known about this process than recruitment of the proteasome [168], and it is not known whether Cdk1 (or its scantily studied interaction partner Cks1) has adaptor functions in other processes as well.

Because considerable attention has been focused on the function of Cdk1 in duplication of the genome (DNA replication, repair and chromosome segregation), the involvement of Cdk1 in other processes associated with the cell cycle is not as well studied, like for instance cell metabolism. When the cell enters the cell cycle, enormous changes take place in catabolic and anabolic processes to facilitate duplication of the genome and biosynthesis of cellular structures and organelles, and therefore one might expect Cdk1 to have a direct role in controlling enzymes required for biosynthesis. However, apart from a few Cdk1 targets, such as Tgl4 and Smp2, which are involved in fatty acid synthesis, and the transcription factor Pho2, which stimulates the expression of genes involved in purine and histidine biosynthesis pathways, little is known about the role of Cdk1 in cell metabolism. It seems likely that additional targets of Cdk1 exist that control metabolic pathways.

Finally, an important aspect of CDKs is their involvement in tumor growth. Like in *S. cerevisiae*, a single CDK (Cdk1, also known as Cdc2) is sufficient to drive the cell cycle in higher eukaryotes, but additional CDKs (Cdk2,4,6) are required for proliferation of specialized tissues and development of the organism [28,570,571]. While CDKs are crucial for growth and development of all eukaryotes, the aberrant activity of these CDKs is well known to underlie tumor growth [28]. Numerous studies have shown that tumor cells evade antigrowth signals. One key inhibitor of the cell cycle is p53, which blocks the cell cycle by inhibiting CDK activity in several ways, one of which is inducing the transcription of p21 [572,573], which binds and inactivates cyclin-CDK complexes. Both p53 and p21 are frequently mutated in human cancers [574], as well as other CKIs such as p16 and p27 [28], and most human tumors aberrantly express cyclin D and cyclin E [28], underscoring the importance of proper control of CDK activity. It is becoming clear that CDKs play an important role in the DNA damage response in *S. cerevisiae *as well as mammalian cells, and treatment of cells with DNA damaging agents while simultaneously inhibiting Cdk1 activity results in extreme cell toxicity in *S. cerevisiae *and human cells [325,515,518,575,576]. Currently, several combination therapies are in clinical trial as cancer chemotherapy [577]. The vast majority of current chemotherapies are based on drugs that induce DNA damage or that inhibit mitosis by targeting microtubules, and these therapies frequently result in serious side effects such as mucositis and myelosuppression, and increase the risk of secondary neoplasms. We believe that unraveling the genetic network of *CDK1 *(i.e. the network of genes that become essential under conditions of reduced Cdk1 activity) might identify novel pathways that can be targeted by combination therapy with CDK inhibitors to induce synthetic lethality of cancer cells, thus contributing to more personalized, less toxic and more efficacious chemotherapy.

## Conclusions

In conclusion, the identification of Cdk1 targets during the past decade has greatly improved our understanding of the molecular mechanism of the cell cycle. Nonetheless, much work still needs to be done because many targets remain to be identified, the exact phosphorylation sites of many known Cdk1 targets have not been mapped and the consequences of these phosphorylations at the molecular often remain elusive. The development of modern genetic screens [567,578] and tools to specifically target Cdk1 activity [579], and the identification of a large collection of potential Cdk1 targets [17,126,580] will catalyze the identification of novel processes and targets controlled by Cdk1. This, and the unraveling of the genetic network of the cell cycle may aid in development of more efficacious cancer chemotherapy.

## List of abbreviations

APC: anaphase promoting complex; BER: base excision repair; BRCT: breast cancer 1 early onset C-terminal region; CAK: cyclin dependent kinase activating kinase; CDK: cyclin dependent kinase; CIN: chromosomal instability; CKI: cyclin dependent kinase inhibitor; DDK: Dbf4 dependent kinase; DSB: DNA double strand break; FEAR: Cdc Fourteen early anaphase release; GAP: GTPase activating protein; GCR: gross chromosomal rearrangement; GEF: guanine nucleotide exchange factor; GIN: genomic instability; HO: homothallic endonuclease; HR: homologous recombination; HU: hydroxyurea; INCENP: inner centromere-like protein; MAP: microtubule-associated protein; MAPK: MAP kinase; MBF: Mlu1-box binding factor; MCB: Mlu1 cell cycle box; MEN: mitotic exit network; MMR: DNA mismatch repair; MMS: methyl methanosulfonate; MTOC: microtubule-organizing center; NER: nucleotide excision repair; NHEJ: non-homologous end-joining; NLS: nuclear localization signal; ORC: origin of replication; PAK: p21-activated kinase; PRE: pheromone response element; PRE-IC: pre-initiation complex; Pre-RC: pre-replication complex; ROS: reactive oxygen species; SBF: SCB binding factor; SCB: Swi4/6 cell cycle box; SCF: Skp: Cullin: F-box containing complex; SDL: synthetic dosage lethality; SFF: SWI Five Factor; SGA: synthetic genetic array; SPB: spindle pole body; SPOC: spindle positioning checkpoint; UV: ultraviolet.

## Competing interests

The authors declare that they have no competing interests.

## Authors' contributions

JME and RDK wrote the manuscript. Both authors have read and approved the final manuscript.

## Authors' information

The research group headed by JME http://www.rr-research.no/enserink is focused on the molecular mechanisms of the cell cycle by identifying novel targets and processes controlled by CDKs using the model organism *S. cerevisiae*. RDK's research group is using *S. cerevisiae *to study the molecular mechanisms by which cells maintain genome stability and prevent the accumulation of mutations and other types of genome rearrangements.

## Supplementary Material

Additional file 1 **Targets of Cdk1**. Description of data: The table lists currently known targets of Cdk1, and includes information on sites phosphorylated by Cdk1. Click here for file
