# Supplementary material for: An overview of Cdk1-controlled targets and processes
Source: Cell Div. 2010 May 13;5:11. doi: 10.1186/1747-1028-5-11 (PMC2876151; doi:10.1186/1747-1028-5-11)
Supplement: Additional file 1 — Targets of Cdk1. Description of data: The table lists currently known targets of Cdk1, and includes information on sites phosphorylated by Cdk1. [file 1747-1028-5-11-S1.PDF]

Additional Table 1. Targets of Cdk1[a].

Common, systematic name	Biological function	Cdk1 phosphorylation sites and consequence of phosphorylation	Reference
Regulation of Cdk1 activity			
Cln1, YMR199W	G1 cyclin	The exact phosphorylation sites have not been mapped, but phosphorylation of Cln1 is thought to target it for degradation by the SCF	[1-3]
Cln2, YPL256C	G1 cyclin	Alanine substitution of T311, T381, S396, T405, S427, T430 and S518 inhibits degradation by the SCF	[1-3]
Cln3, YAL040C	G1 cyclin	Alanine substitution of S468 prevents degradation by the SCF, but other phosphorylation sites may exist	[2, 4, 5]
Clb6, YPR119W	S cyclin	The exact phosphorylation sites have not been mapped, but phosphorylation by Cdk1 (and Pho85) is thought to target Clb6 for destruction by the SCF	[6]
Far1, YJL157C	CKI	Phosphorylation of S87 targets Far1 for degradation	[7]
Sic1, YLR079W	CKI	Sic1 harbors nine Cdk1 consensus sites (T2, T5, T33, T45, S69, S76, S80, T173, and S191) and phosphorylation of at least six of these sites targets Sic1 for degradation	[3, 8-10]
Swe1, YJL187C	Protein kinase	Phosphorylation of T45, S56[b], S63[b], T74[b], S105[b], S111, T121, T124, S127[b], S133, S136[b], T196, S201, S263, S266[b], T367[b], T373, T384[b] depends on Cdk1. Alanine substitution of all these sites resulted in	[11, 12]

		reduced kinase activity of Swe1. In addition, phosphorylation of Swe1 by Cdk1 on unknown residues is involved in its degradation	
Mih1, YMR036C	Protein phosphatase	Phosphorylation sites have not been mapped, and the consequences of phosphorylation are not clear, but probably affect the phosphatase activity towards Y19 of Cdk1.	[13]
DNA replication			
Sld2, YKL108W	Replication factor	Cdk1 phosphorylates Sld2 on a number of residues, but especially phosphorylation of T84 stimulates binding to Dpb11 and contributes to initiation of DNA replication	[14-17]
Sld3, YGL113W	Replication factor	Alanine substitution of T600 and S622 is lethal; phosphorylation of Sld3 stimulates binding to Dpb11 and initiation of replication	[14, 16]
Pol12, YBL035C	Alpha-primase complex	Phosphorylation sites have not been mapped, but phosphorylation of Pol12 is thought to inhibit its interaction with chromatin	[18, 19]
Cdc6, YJL194W	Replication factor	Simultaneous alanine substitution of T7, T23 and S43 stabilizes Cdc6 by preventing degradation by the SCF. Alanine substitution of T134 and S372 also stabilizes Cdc6, possibly by preventing degradation by the APC	[20]
Orc2, YBR060C	Replication factor	Orc2 contains six optimal Cdk1 sites (S16, T24, T70, T174, S188, S206), but *in vivo* phosphorylation sites have not been mapped, except S188, the phosphorylation of which may inhibit ATP binding by Orc5	[21]

Orc6, YHR118C	Replication factor	Orc6 contains four optimal Cdk1 sites (S106, S116, S123, T146), but exact phosphorylation sites have not been mapped; alanine substitution of Cdk1sites has revealed that phosphorylation of Orc6 is important for prevention of re-replication	[21, 22]
Mcm3, YEL032W	Replication factor	Phosphorylation of at least 5 Cdk1 sites in the NLS portion of Mcm3 results in nuclear export of the entire Mcm2-7 complex	[23]
Dpb2, YPR175W	Replication factor	The main phosphorylation site is thought to be S144, and alanine substitution of S144 in combination with S125 and S616 reduces the interaction between Dpb2 and Pol2	[22, 24]
Anaphase promoting complex			
Cdh1, YGL003C	APC activator	Simultaneous alanine substitution of S16, S42, T157 and T173 results in defects in SPB separation; phosphorylation of S16, S42, S227, S239 and S436, and T176 abolishes the interaction between Hct1 and APC, thus inhibiting APC activation	[25-27]
Acm1, YPL267W	Cdh1 inhibitor	Phosphorylation of S37, S48, S102, S202 (not a Cdk1 consensus site) and S161 may stabilize Acm1, protecting it from proteasome-mediated destruction; alanine substitution of the full CDK consensus sites (S3, S31, S48, S102, T161) results in nuclear accumulation and enhanced degradation of Acm1	[28-30]
Cdc16, YKL022C	APC component	Alanine substitution of S44, S59, S95, S103, T115, T406 (*cdc16-6A* allele) inhibits binding of Cdc20 to the APC and thereby APC activation, leading to mitotic delay	[31]

Cdc23, YHR166C	APC component	Alanine substitution of the minimal Cdk1 site (S59) exacerbated the defect of *cdc16-6A* and *cdc27-5A* mutants	[31]
Cdc27, YBL084C	APC component	Alanine substitution of the Cdk1 sites S267, T304, S328, T351, T397 (the *cdc27-5A* allele) prevents Cks1 binding to the APC and leads to mitotic delay	[31]
Pheromone signaling			
Ste20, YHL007C	PAK family kinase	The exact Cdk1 phosphorylation sites have not been mapped, but phosphorylation of Ste20 by Cdk1 may inhibit Ste20, thus restricting pheromone signaling to the G1 phase of the cell cycle	[32, 33]
Ste5, YDR103W	Adaptor	Alanine substitution of 8 Cdk1 sites (T4, S11, T29, S43, S69, S71, S81, T102) triggers aberrant pheromone arrest outside G1, possibly because phosphorylation by Cln-Cdk1 promotes SCF-mediated degradation of Ste5	[34, 35]
Transcription			
Ace2, YLR131C	Transcription factor	Alanine substitution of T575, S701 and S714 results in constitutive nuclear localization of Ace2	[36, 37]
Smp2, YMR165C	Transcription inhibitor	Alanine substitution of S110, S114, S168, S602, T723, S744, S748 results in derepression of transcription of ER-localized phospholipid biosynthesis enzymes, which may support nuclear membrane synthesis	[38, 39]
Whi5, YOR083W	Transcription inhibitor	Whi5 is phosphorylated on at least 16 sites *in vivo* (10 Cdk1 consensus sites and 6 non-consensus sites); phosphorylation of Whi5 induces release from SBF and exclusion from the nucleus	[40-42]

Swi6, YLR182W	Transcription factor	Phosphorylation of S160 promotes nuclear export	[43]
Swi5, YDR146C	Transcription factor	Phosphorylation of S646 and S664 promotes nuclear export	[44]
Fkh2, YNL068C	Transcription factor	Phosphorylation of S683 and T697 promotes the interaction with Ndd1 to activate transcription	[45]
Ndd1, YOR372C	Transcription factor	Phosphorylation of T319 enhances the interaction with Fkh2, thus promoting transcription	[46, 47]
Stb1, YNL309W	Transcription factor	The exact phosphorylation sites have not been mapped, but phosphorylation by Cln-Cdk1 may abrogate the interaction between Stb1 and Swi6 and may release Stb1 from promoter regions	[48-50]
Pho2, YDL106C	Transcription factor	Phosphorylation of S230 promotes the interaction between Pho2 and Pho4 to induce transcription	[51]
Msa1, YOR066W	Transcription factor	Alanine substitution of S54 and T84 results in its nuclear localization	[52]
Initiation of anaphase, FEAR, MEN			
Pds1, YDR113C	Securin	S37, S71, S212, S278, S292, S304 are phosphorylated *in vivo*; phosphorylation of both S37 and S71 inhibits degradation by degradation by the APC, wile phosphorylation of S212, S278 and S304 is thought to be important for the interaction between Pds1 and Esp1 and nuclear import of the Pds1-Esp1 complex	[53, 54]
Cdc5, YMR001C	Polo kinase	Phosphorylation of T242 contributes to activation of Cdc5	[55]

Net1 (Cfi1), YJL076W	RENT complex	Net1 is phosphorylated *in vivo* on 19 sites, and the *net1-3cdk* mutant carrying alanine substitution of 3 Cdk1 consensus sites (S166, T212, S252) fails to release Cdc14	[56]
Spo12, YHR152W	Nucleolar protein	Alanine substitution of S118 and S125 results in mitotic delay, but also in synthetic lethality when combined with an *lte1Δ* mutation	[57]
Lte1, YAL024C	Putative GEF	The exact phosphorylation sites have not been mapped, but Lte1 phosphorylation by Cdk1 is thought to be required for its localization to the bud cortex, although a mutant with alanine substitution of five (T317, T614, S630, S667, T793) out of a total of nine potential Cdk1 sites displayed no phenotype	[58]
Cdc15, YAR019C	Protein kinase	Alanine substitution of 7 Cdk1 consensus sites results in more robust mitotic exit without affecting kinase activity	[59]
DNA repair, checkpoint, telomere maintenance			
Srs2, YJL092W	DNA helicase	Alanine substitution of seven Cdk1 consensus sites (T604, S698, S879, S938, S893, S950, and S965) leads to defects in damage-induced formation of Sgs1-Mre11 and Srs2-Mre11 subcomplexes and sensitivity to MMS, and inefficient DNA repair	[60-62]
Dna2	Nuclease	Phosphorylation in its NLS sequence results in nuclear import	[52]
Sae2, YGL175C	Nuclease	Phosphorylation of S267 increases Sae2 nuclease activity to resect DSBs	[63]
Yen1,	Holliday	Alanine substitution of S679 results in its nuclear	[52]

YER041W	junction resolvase	accumulation	
Cdc13, YDL220C	Telomere capping	Phosphorylation of T308 recruits the telomerase complex to telomeres resulting in telomere elongation; may also promote subsequent phosphorylation of S306 by Mec1. T336 is also phosphorylated, but the consequence remains unknown	[64, 65]
Mer2, YJR021C	Meiotic DSB formation	Phosphorylation of S30 and S271 promotes Spo11-mediated formation of DSBs at meiotic recombination hotspots	[66-68]
Rad9, YDR217C	Adaptor	Alanine substitution of 18 N-terminal sites reduces Rad53 activation under low zeocin concentrations, but has no effect on Rad53 activation at high zeocin concentrations	[69]
Rad53, YPL153C	Checkpoint kinase similar to mammalian Chk2	Aspartate substitution of S774 results in increased sensitivity to calcofluor white, indicating an involvement of Rad53 in cell morphogenesis; alanine substitution of S774 resulted in enhanced checkpoint adaptation in the presence of DNA damage	[70, 71]
Psy4, YBL046W	Regulatory subunit of a phosphatase involved in the repair of cross-linked DNA	Alanine substitution of T320 results in its nuclear localization	[52]
Regulation of SPBs, kinetochores, mitotic spindle			
Ase1,	Microtubule	T55, S64, S198, T675, S707, S803, S819 are potential	[22, 72-74]

YOR058C	associated protein	phosphorylation sites, and dephosphorylation of Ase1 is involved in spindle midzone organization	
Ask1, YKL052C	DASH complex	Alanine substitution of S216 and S250 exacerbates temperature-sensitivity when combined with the additional mutation of a temperature-sensitive *ask1-3* allele	[75]
Bir1, YJR089W	Passenger complex	Alanine substitution of nine sites (S383A, S395A, S552A, S587A, S667A, T684A, S688A, T735A, and T747A) prevents localization of Ndc10 to the spindle at anaphase	[76]
Cnm67, YNL225C	SPB outer plaque	Phosphorylation of Cnm67 may contribute to asymmetric localization of dynein	[77]
Fin1, YDR130C	Intermediate filament protein	Alanine substitution of the full CDK consensus sites (S36, S54, T68, S117, S148) localizes Fin1 to the spindle before anaphase and impairs efficient chromosome segregation	[22, 78]
Kar9, YPL269W	Karyogamy protein	Phosphorylation of S197 and S496 contributes to asymmetric loading of Kar9 onto SPBs	[79, 80]
Mps1, YDL028C	Protein kinase	Phosphorylation of T29 stabilizes Mps1 and contributes to SPB duplication	[81]
Nud1, YOR373W	SPB outer plaque	Unknown	[82]
Sli15, YBR156C	Passenger complex	Alanine substitution of S335, S373, S427, S437, S462 and T474 results in aberrant localization of Sli15-Ipl1 to the mitotic spindle	[83]

Slk19, YOR195W	Kinetochore protein	Unknown	[22, 82]
Spc110, YDR356W	SPB inner plaque	Phosphorylation at position 91 may be important for spindle integrity and mitotic spindle elongation; S36 may also be phosphorylated, but the function is unknown	[84]
Spc42, YKL042W	SPB central plaque	Phosphorylation on S4 and T6 mediates SPB duplication	[81]
Stu2, YLR045C	Microtubule associated protein	Unknown	[82]
Cell polarity, budding, organelle inheritance			
Bem1, YBR200W	Adaptor	Phosphorylation of S72 promotes vacuole homeostasis	[85]
Bem2, YER155C	GAP	Phosphorylation sites have not been mapped. Based on similarity to Bem3, phosphorylation probably inhibits GAP activity	[86]
Bem3, YPL115C	GAP	Bem3 is phosphorylated on S195, S222, S254, S880 and possibly S585, which may inhibit GAP activity, thus contributing to actin polarization and bud emergence	[86]
Boi1, YBL085W	Adaptor	Boi1 is phosphorylated on S86, S149, T158, S229, S393, S405, S412, S540, S605, T670, T909; additional, non-Cdk1 consensus sites are also phosphorylated, potentially by Cdk1. Simultaneous alanine substitution results in defects in polarized cell	[87]

		growth	
Boi2, YER114C	Adaptor	Phosphorylation sites have not been mapped and no alanine substitutions of Cdk1 consensus sites have been made; based on homology to Boi1, phosphorylation of Boi2 may be involved in establishment/maintenance of cell polarity	[87]
Cdc3, YLR314C	Septin	Phosphorylation of S503 and S509 may be involved in disassembly of the old septin ring	[88]
Rga2, YDR379W	GAP	Alanine substitution of S380 in combination with S334, S692, S707, S733, S763, S770, and S772 results in cell polarity defects	[87, 89]
Shs1, YDL225W	Septin	Phosphorylation on several sites (T6, T386, S416, S441, S447) by either Cdk1 or Pho85 enhances Cdk1 inhibitory phosphorylation of Cdk1 by Swe1 later in the cell cycle	[90]
Tgl4, YKR089C	Lipid phosphatase	Phosphorylation on T675 and S890 activates its lipase activity, which mobilizes fatty acids to support initiation of bud formation in late G1	[22, 91]
Tus1, YLR425W	GEF	Simultaneous alanine substitution of S8, T13, T93, S122, S126, S163, S170, S173, S176 (all minimal Cdk1 sites), or alanine substitution of T13 and S135 (both full Cdk1 consensus sites) leads to decreased Rho1 activity and actin polymerization defects	[92]
Vac17, YCL063W	Adaptor	Alanine substitution of T149, S178, S119 and T248 results in defects in vacuole inheritance	[93]

Other			
Chs2, YBR038W	Chitin synthase	Glutamate substitutions of the full Cdk1 consensus sites S14, S60, S69 and S100 resulted in constitutive ER retention of Chs2, while alanine substitutions lead to constitutive ER export	[94]
Nup53, YMR153W	Nucleoporin	Simultaneous alanine substitution of the Cdk1 consensus sites S101 and S206 ameliorated the growth defect of *nup170Δ* mutants, possibly by modulating the spindle assembly checkpoint	[95]

[a] Included in this table are proteins for which Cdk1-mediated phosphorylation has been studied in at least some detail. As indicated by large-scale proteomic studies, it is likely that many more *bona fide* Cdk1 targets exist [22, 96, 97], however these potential Cdk1 substrates still await more detailed characterization to establish that they are indeed directly phosphorylated by Cdk1 *in vivo*.

[b] Does not conform to the minimal Cdk1 consensus site (i.e. SP/TP).

References

1. Lanker S, Valdivieso MH, Wittenberg C: **Rapid degradation of the G1 cyclin Cln2 induced by CDK-dependent phosphorylation.** *Science* 1996, **271:**1597-1601.
2. Willems AR, Lanker S, Patton EE, Craig KL, Nason TF, Mathias N, Kobayashi R, Wittenberg C, Tyers M: **Cdc53 targets phosphorylated G1 cyclins for degradation by the ubiquitin proteolytic pathway.** *Cell* 1996, **86:**453-463.
3. Skowyra D, Craig KL, Tyers M, Elledge SJ, Harper JW: **F-box proteins are receptors that recruit phosphorylated substrates to the SCF ubiquitin-ligase complex.** *Cell* 1997, **91:**209-219.
4. Tyers M, Tokiwa G, Nash R, Futcher B: **The Cln3-Cdc28 kinase complex of S. cerevisiae is regulated by proteolysis and phosphorylation.** *EMBO J* 1992, **11:**1773-1784.
5. Yaglom J, Linskens MH, Sadis S, Rubin DM, Futcher B, Finley D: **p34Cdc28-mediated control of Cln3 cyclin degradation.** *Mol Cell Biol* 1995, **15:**731-741.
6. Jackson LP, Reed SI, Haase SB: **Distinct mechanisms control the stability of the related S-phase cyclins Clb5 and Clb6.** *Mol Cell Biol* 2006, **26:**2456-2466.
7. Henchoz S, Chi Y, Catarin B, Herskowitz I, Deshaies RJ, Peter M: **Phosphorylation- and ubiquitin-dependent degradation of the cyclin-dependent kinase inhibitor Far1p in budding yeast.** *Genes Dev* 1997, **11:**3046-3060.

8. Verma R, Annan RS, Huddleston MJ, Carr SA, Reynard G, Deshaies RJ: **Phosphorylation of Sic1p by G1 Cdk required for its degradation and entry into S phase.** *Science* 1997, **278:**455-460.
9. Nash P, Tang X, Orlicky S, Chen Q, Gertler FB, Mendenhall MD, Sicheri F, Pawson T, Tyers M: **Multisite phosphorylation of a CDK inhibitor sets a threshold for the onset of DNA replication.** *Nature* 2001, **414:**514-521.
10. Schneider BL, Yang QH, Futcher AB: **Linkage of replication to start by the Cdk inhibitor Sic1.** *Science* 1996, **272:**560-562.
11. Harvey SL, Charlet A, Haas W, Gygi SP, Kellogg DR: **Cdk1-dependent regulation of the mitotic inhibitor Wee1.** *Cell* 2005, **122:**407-420.
12. Asano S, Park JE, Sakchaisri K, Yu LR, Song S, Supavilai P, Veenstra TD, Lee KS: **Concerted mechanism of Swe1/Wee1 regulation by multiple kinases in budding yeast.** *Embo J* 2005, **24:**2194-2204.
13. Pal G, Paraz MT, Kellogg DR: **Regulation of Mih1/Cdc25 by protein phosphatase 2A and casein kinase 1.** *J Cell Biol* 2008, **180:**931-945.
14. Zegerman P, Diffley JF: **Phosphorylation of Sld2 and Sld3 by cyclin-dependent kinases promotes DNA replication in budding yeast.** *Nature* 2007, **445:**281-285.
15. Tak YS, Tanaka Y, Endo S, Kamimura Y, Araki H: **A CDK-catalysed regulatory phosphorylation for formation of the DNA replication complex Sld2-Dpb11.** *EMBO J* 2006, **25:**1987-1996.
16. Tanaka S, Umemori T, Hirai K, Muramatsu S, Kamimura Y, Araki H: **CDK-dependent phosphorylation of Sld2 and Sld3 initiates DNA replication in budding yeast.** *Nature* 2007, **445:**328-332.
17. Masumoto H, Muramatsu S, Kamimura Y, Araki H: **S-Cdk-dependent phosphorylation of Sld2 essential for chromosomal DNA replication in budding yeast.** *Nature* 2002, **415:**651-655.
18. Foiani M, Liberi G, Lucchini G, Plevani P: **Cell cycle-dependent phosphorylation and dephosphorylation of the yeast DNA polymerase alpha-primase B subunit.** *Mol Cell Biol* 1995, **15:**883-891.
19. Desdouets C, Santocanale C, Drury LS, Perkins G, Foiani M, Plevani P, Diffley JF: **Evidence for a Cdc6p-independent mitotic resetting event involving DNA polymerase alpha.** *EMBO J* 1998, **17:**4139-4146.
20. Elsasser S, Chi Y, Yang P, Campbell JL: **Phosphorylation controls timing of Cdc6p destruction: A biochemical analysis.** *Mol Biol Cell* 1999, **10:**3263-3277.
21. Nguyen VQ, Co C, Li JJ: **Cyclin-dependent kinases prevent DNA re-replication through multiple mechanisms.** *Nature* 2001, **411:**1068-1073.
22. Ubersax JA, Woodbury EL, Quang PN, Paraz M, Blethrow JD, Shah K, Shokat KM, Morgan DO: **Targets of the cyclin-dependent kinase Cdk1.** *Nature* 2003, **425:**859-864.
23. Liku ME, Nguyen VQ, Rosales AW, Irie K, Li JJ: **CDK phosphorylation of a novel NLS-NES module distributed between two subunits of the Mcm2-7 complex prevents chromosomal rereplication.** *Mol Biol Cell* 2005, **16:**5026-5039.
24. Kesti T, McDonald WH, Yates JR, 3rd, Wittenberg C: **Cell cycle-dependent phosphorylation of the DNA polymerase epsilon subunit, Dpb2, by the Cdc28 cyclin-dependent protein kinase.** *J Biol Chem* 2004, **279:**14245-14255.

25. Crasta K, Lim HH, Giddings TH, Jr., Winey M, Surana U: **Inactivation of Cdh1 by synergistic action of Cdk1 and polo kinase is necessary for proper assembly of the mitotic spindle.** *Nat Cell Biol* 2008, **10:**665-675.
26. Zachariae W, Schwab M, Nasmyth K, Seufert W: **Control of cyclin ubiquitination by CDK-regulated binding of Hct1 to the anaphase promoting complex.** *Science* 1998, **282:**1721-1724.
27. Jaspersen SL, Charles JF, Morgan DO: **Inhibitory phosphorylation of the APC regulator Hct1 is controlled by the kinase Cdc28 and the phosphatase Cdc14.** *Curr Biol* 1999, **9:**227-236.
28. Hall MC, Jeong DE, Henderson JT, Choi E, Bremmer SC, Iliuk AB, Charbonneau H: **Cdc28 and Cdc14 control stability of the anaphase-promoting complex inhibitor Acm1.** *J Biol Chem* 2008, **283:**10396-10407.
29. Enquist-Newman M, Sullivan M, Morgan DO: **Modulation of the mitotic regulatory network by APC-dependent destruction of the Cdh1 inhibitor Acm1.** *Mol Cell* 2008, **30:**437-446.
30. Ostapenko D, Burton JL, Wang R, Solomon MJ: **Pseudosubstrate inhibition of the anaphase-promoting complex by Acm1: regulation by proteolysis and Cdc28 phosphorylation.** *Mol Cell Biol* 2008, **28:**4653-4664.
31. Rudner AD, Murray AW: **Phosphorylation by Cdc28 activates the Cdc20-dependent activity of the anaphase-promoting complex.** *J Cell Biol* 2000, **149:**1377-1390.
32. Wu C, Leeuw T, Leberer E, Thomas DY, Whiteway M: **Cell cycle- and Cln2p-Cdc28p-dependent phosphorylation of the yeast Ste20p protein kinase.** *J Biol Chem* 1998, **273:**28107-28115.
33. Oehlen LJ, Cross FR: **Potential regulation of Ste20 function by the Cln1-Cdc28 and Cln2-Cdc28 cyclin-dependent protein kinases.** *J Biol Chem* 1998, **273:**25089-25097.
34. Garrenton LS, Braunwarth A, Irniger S, Hurt E, Kunzler M, Thorner J: **Nucleus-specific and cell cycle-regulated degradation of mitogen-activated protein kinase scaffold protein Ste5 contributes to the control of signaling competence.** *Mol Cell Biol* 2009, **29:**582-601.
35. Strickfaden SC, Winters MJ, Ben-Ari G, Lamson RE, Tyers M, Pryciak PM: **A mechanism for cell-cycle regulation of MAP kinase signaling in a yeast differentiation pathway.** *Cell* 2007, **128:**519-531.
36. O'Conallain C, Doolin MT, Taggart C, Thornton F, Butler G: **Regulated nuclear localisation of the yeast transcription factor Ace2p controls expression of chitinase (CTS1) in Saccharomyces cerevisiae.** *Mol Gen Genet* 1999, **262:**275-282.
37. Archambault V, Chang EJ, Drapkin BJ, Cross FR, Chait BT, Rout MP: **Targeted proteomic study of the cyclin-Cdk module.** *Mol Cell* 2004, **14:**699-711.
38. Santos-Rosa H, Leung J, Grimsey N, Peak-Chew S, Siniossoglou S: **The yeast lipin Smp2 couples phospholipid biosynthesis to nuclear membrane growth.** *EMBO J* 2005, **24:**1931-1941.
39. O'Hara L, Han GS, Peak-Chew S, Grimsey N, Carman GM, Siniossoglou S: **Control of phospholipid synthesis by phosphorylation of the yeast lipin Pah1p/Smp2p**

Mg2+-dependent phosphatidate phosphatase. *J Biol Chem* 2006, **281:**34537-34548.

40. de Bruin RA, McDonald WH, Kalashnikova TI, Yates J, 3rd, Wittenberg C: **Cln3 activates G1-specific transcription via phosphorylation of the SBF bound repressor Whi5.** *Cell* 2004, **117:**887-898.
41. Costanzo M, Nishikawa JL, Tang X, Millman JS, Schub O, Breitkreuz K, Dewar D, Rupes I, Andrews B, Tyers M: **CDK activity antagonizes Whi5, an inhibitor of G1/S transcription in yeast.** *Cell* 2004, **117:**899-913.
42. Wagner MV, Smolka MB, de Bruin RA, Zhou H, Wittenberg C, Dowdy SF: **Whi5 regulation by site specific CDK-phosphorylation in Saccharomyces cerevisiae.** *PLoS ONE* 2009, **4:**e4300.
43. Geymonat M, Spanos A, Wells GP, Smerdon SJ, Sedgwick SG: **Clb6/Cdc28 and Cdc14 regulate phosphorylation status and cellular localization of Swi6.** *Mol Cell Biol* 2004, **24:**2277-2285.
44. Moll T, Tebb G, Surana U, Robitsch H, Nasmyth K: **The role of phosphorylation and the CDC28 protein kinase in cell cycle-regulated nuclear import of the S. cerevisiae transcription factor SWI5.** *Cell* 1991, **66:**743-758.
45. Pic-Taylor A, Darieva Z, Morgan BA, Sharrocks AD: **Regulation of cell cycle-specific gene expression through cyclin-dependent kinase-mediated phosphorylation of the forkhead transcription factor Fkh2p.** *Mol Cell Biol* 2004, **24:**10036-10046.
46. Darieva Z, Pic-Taylor A, Boros J, Spanos A, Geymonat M, Reece RJ, Sedgwick SG, Sharrocks AD, Morgan BA: **Cell cycle-regulated transcription through the FHA domain of Fkh2p and the coactivator Ndd1p.** *Curr Biol* 2003, **13:**1740-1745.
47. Reynolds D, Shi BJ, McLean C, Katsis F, Kemp B, Dalton S: **Recruitment of Thr 319-phosphorylated Ndd1p to the FHA domain of Fkh2p requires Clb kinase activity: a mechanism for CLB cluster gene activation.** *Genes Dev* 2003, **17:**1789-1802.
48. Ho Y, Costanzo M, Moore L, Kobayashi R, Andrews BJ: **Regulation of transcription at the Saccharomyces cerevisiae start transition by Stb1, a Swi6-binding protein.** *Mol Cell Biol* 1999, **19:**5267-5278.
49. de Bruin RA, Kalashnikova TI, Wittenberg C: **Stb1 collaborates with other regulators to modulate the G1-specific transcriptional circuit.** *Mol Cell Biol* 2008, **28:**6919-6928.
50. Costanzo M, Schub O, Andrews B: **G1 transcription factors are differentially regulated in Saccharomyces cerevisiae by the Swi6-binding protein Stb1.** *Mol Cell Biol* 2003, **23:**5064-5077.
51. Liu C, Yang Z, Yang J, Xia Z, Ao S: **Regulation of the yeast transcriptional factor PHO2 activity by phosphorylation.** *J Biol Chem* 2000, **275:**31972-31978.
52. Kosugi S, Hasebe M, Tomita M, Yanagawa H: **Systematic identification of cell cycle-dependent yeast nucleocytoplasmic shuttling proteins by prediction of composite motifs.** *Proc Natl Acad Sci U S A* 2009, **106:**10171-10176.
53. Holt LJ, Krutchinsky AN, Morgan DO: **Positive feedback sharpens the anaphase switch.** *Nature* 2008, **454:**353-357.

54. Agarwal R, Cohen-Fix O: **Phosphorylation of the mitotic regulator Pds1/securin by Cdc28 is required for efficient nuclear localization of Esp1/separase.** *Genes Dev* 2002, **16:**1371-1382.
55. Mortensen EM, Haas W, Gygi M, Gygi SP, Kellogg DR: **Cdc28-dependent regulation of the Cdc5/Polo kinase.** *Curr Biol* 2005, **15:**2033-2037.
56. Azzam R, Chen SL, Shou W, Mah AS, Alexandru G, Nasmyth K, Annan RS, Carr SA, Deshaies RJ: **Phosphorylation by cyclin B-Cdk underlies release of mitotic exit activator Cdc14 from the nucleolus.** *Science* 2004, **305:**516-519.
57. Tomson BN, Rahal R, Reiser V, Monje-Casas F, Mekhail K, Moazed D, Amon A: **Regulation of Spo12 phosphorylation and its essential role in the FEAR network.** *Curr Biol* 2009, **19:**449-460.
58. Jensen S, Geymonat M, Johnson AL, Segal M, Johnston LH: **Spatial regulation of the guanine nucleotide exchange factor Lte1 in Saccharomyces cerevisiae.** *J Cell Sci* 2002, **115:**4977-4991.
59. Jaspersen SL, Morgan DO: **Cdc14 activates cdc15 to promote mitotic exit in budding yeast.** *Curr Biol* 2000, **10:**615-618.
60. Chiolo I, Carotenuto W, Maffioletti G, Petrini JH, Foiani M, Liberi G: **Srs2 and Sgs1 DNA helicases associate with Mre11 in different subcomplexes following checkpoint activation and CDK1-mediated Srs2 phosphorylation.** *Mol Cell Biol* 2005, **25:**5738-5751.
61. Liberi G, Chiolo I, Pellicioli A, Lopes M, Plevani P, Muzi-Falconi M, Foiani M: **Srs2 DNA helicase is involved in checkpoint response and its regulation requires a functional Mec1-dependent pathway and Cdk1 activity.** *Embo J* 2000, **19:**5027-5038.
62. Saponaro M, Callahan D, Zheng X, Krejci L, Haber JE, Klein HL, Liberi G: **Cdk1 targets Srs2 to complete synthesis-dependent strand annealing and to promote recombinational repair.** *PLoS Genet*, **6:**e1000858.
63. Huertas P, Cortes-Ledesma F, Sartori AA, Aguilera A, Jackson SP: **CDK targets Sae2 to control DNA-end resection and homologous recombination.** *Nature* 2008, **455:**689-692.
64. Li S, Makovets S, Matsuguchi T, Blethrow JD, Shokat KM, Blackburn EH: **Cdk1-dependent phosphorylation of Cdc13 coordinates telomere elongation during cell-cycle progression.** *Cell* 2009, **136:**50-61.
65. Tseng SF, Shen ZJ, Tsai HJ, Lin YH, Teng SC: **Rapid Cdc13 turnover and telomere length homeostasis are controlled by Cdk1-mediated phosphorylation of Cdc13.** *Nucleic Acids Res* 2009, **37:**3602-3611.
66. Henderson KA, Kee K, Maleki S, Santini PA, Keeney S: **Cyclin-dependent kinase directly regulates initiation of meiotic recombination.** *Cell* 2006, **125:**1321-1332.
67. Wan L, Niu H, Futcher B, Zhang C, Shokat KM, Boulton SJ, Hollingsworth NM: **Cdc28-Clb5 (CDK-S) and Cdc7-Dbf4 (DDK) collaborate to initiate meiotic recombination in yeast.** *Genes Dev* 2008, **22:**386-397.
68. Sasanuma H, Hirota K, Fukuda T, Kakusho N, Kugou K, Kawasaki Y, Shibata T, Masai H, Ohta K: **Cdc7-dependent phosphorylation of Mer2 facilitates initiation of yeast meiotic recombination.** *Genes Dev* 2008, **22:**398-410.

69. Bonilla CY, Melo JA, Toczyski DP: **Colocalization of sensors is sufficient to activate the DNA damage checkpoint in the absence of damage.** *Mol Cell* 2008, **30:**267-276.
70. Diani L, Colombelli C, Nachimuthu BT, Donnianni R, Plevani P, Muzi-Falconi M, Pellicioli A: **Saccharomyces CDK1 phosphorylates Rad53 kinase in metaphase, influencing cellular morphogenesis.** *J Biol Chem* 2009, **284:**32627-32634.
71. Schleker T, Shimada K, Sack R, Pike BL, Gasser SM: **Cell cycle-dependent phosphorylation of Rad53 kinase by Cdc5 and Cdc28 modulates checkpoint adaptation.** *Cell Cycle*, **9:**350-363.
72. Jin F, Liu H, Liang F, Rizkallah R, Hurt MM, Wang Y: **Temporal control of the dephosphorylation of Cdk substrates by mitotic exit pathways in budding yeast.** *Proc Natl Acad Sci U S A* 2008, **105:**16177-16182.
73. Crasta K, Huang P, Morgan G, Winey M, Surana U: **Cdk1 regulates centrosome separation by restraining proteolysis of microtubule-associated proteins.** *EMBO J* 2006, **25:**2551-2563.
74. Khmelinskii A, Lawrence C, Roostalu J, Schiebel E: **Cdc14-regulated midzone assembly controls anaphase B.** *J Cell Biol* 2007, **177:**981-993.
75. Li Y, Elledge SJ: **The DASH complex component Ask1 is a cell cycle-regulated Cdk substrate in Saccharomyces cerevisiae.** *Cell Cycle* 2003, **2:**143-148.
76. Widlund PO, Lyssand JS, Anderson S, Niessen S, Yates JR, 3rd, Davis TN: **Phosphorylation of the chromosomal passenger protein Bir1 is required for localization of Ndc10 to the spindle during anaphase and full spindle elongation.** *Mol Biol Cell* 2006, **17:**1065-1074.
77. Grava S, Schaerer F, Faty M, Philippsen P, Barral Y: **Asymmetric recruitment of dynein to spindle poles and microtubules promotes proper spindle orientation in yeast.** *Dev Cell* 2006, **10:**425-439.
78. Woodbury EL, Morgan DO: **Cdk and APC activities limit the spindle-stabilizing function of Fin1 to anaphase.** *Nat Cell Biol* 2007, **9:**106-112.
79. Moore JK, D'Silva S, Miller RK: **The CLIP-170 homologue Bik1p promotes the phosphorylation and asymmetric localization of Kar9p.** *Mol Biol Cell* 2006, **17:**178-191.
80. Liakopoulos D, Kusch J, Grava S, Vogel J, Barral Y: **Asymmetric loading of Kar9 onto spindle poles and microtubules ensures proper spindle alignment.** *Cell* 2003, **112:**561-574.
81. Jaspersen SL, Huneycutt BJ, Giddings TH, Jr., Resing KA, Ahn NG, Winey M: **Cdc28/Cdk1 regulates spindle pole body duplication through phosphorylation of Spc42 and Mps1.** *Dev Cell* 2004, **7:**263-274.
82. Park CJ, Park JE, Karpova TS, Soung NK, Yu LR, Song S, Lee KH, Xia X, Kang E, Dabanoglu I, et al: **Requirement for the budding yeast polo kinase Cdc5 in proper microtubule growth and dynamics.** *Eukaryot Cell* 2008, **7:**444-453.
83. Pereira G, Schiebel E: **Separase regulates INCENP-Aurora B anaphase spindle function through Cdc14.** *Science* 2003, **302:**2120-2124.
84. Huisman SM, Smeets MF, Segal M: **Phosphorylation of Spc110p by Cdc28p-Clb5p kinase contributes to correct spindle morphogenesis in S. cerevisiae.** *J Cell Sci* 2007, **120:**435-446.

85. Han BK, Bogomolnaya LM, Totten JM, Blank HM, Dangott LJ, Polymenis M: **Bem1p, a scaffold signaling protein, mediates cyclin-dependent control of vacuolar homeostasis in Saccharomyces cerevisiae.** *Genes Dev* 2005, **19:**2606-2618.
86. Knaus M, Pelli-Gulli MP, van Drogen F, Springer S, Jaquenoud M, Peter M: **Phosphorylation of Bem2p and Bem3p may contribute to local activation of Cdc42p at bud emergence.** *EMBO J* 2007, **26:**4501-4513.
87. McCusker D, Denison C, Anderson S, Egelhofer TA, Yates JR, 3rd, Gygi SP, Kellogg DR: **Cdk1 coordinates cell-surface growth with the cell cycle.** *Nat Cell Biol* 2007, **9:**506-515.
88. Tang CS, Reed SI: **Phosphorylation of the septin cdc3 in g1 by the cdc28 kinase is essential for efficient septin ring disassembly.** *Cell Cycle* 2002, **1:**42-49.
89. Sopko R, Huang D, Smith JC, Figeys D, Andrews BJ: **Activation of the Cdc42p GTPase by cyclin-dependent protein kinases in budding yeast.** *EMBO J* 2007, **26:**4487-4500.
90. Egelhofer TA, Villen J, McCusker D, Gygi SP, Kellogg DR: **The septins function in G1 pathways that influence the pattern of cell growth in budding yeast.** *PLoS ONE* 2008, **3:**e2022.
91. Kurat CF, Wolinski H, Petschnigg J, Kaluarachchi S, Andrews B, Natter K, Kohlwein SD: **Cdk1/Cdc28-dependent activation of the major triacylglycerol lipase Tgl4 in yeast links lipolysis to cell-cycle progression.** *Mol Cell* 2009, **33:**53-63.
92. Kono K, Nogami S, Abe M, Nishizawa M, Morishita S, Pellman D, Ohya Y: **G1/S cyclin-dependent kinase regulates small GTPase Rho1p through phosphorylation of RhoGEF Tus1p in Saccharomyces cerevisiae.** *Mol Biol Cell* 2008, **19:**1763-1771.
93. Peng Y, Weisman LS: **The cyclin-dependent kinase Cdk1 directly regulates vacuole inheritance.** *Dev Cell* 2008, **15:**478-485.
94. Teh EM, Chai CC, Yeong FM: **Retention of Chs2p in the ER requires N-terminal CDK1-phosphorylation sites.** *Cell Cycle* 2009, **8:**2964-2974.
95. Lusk CP, Waller DD, Makhnevych T, Dienemann A, Whiteway M, Thomas DY, Wozniak RW: **Nup53p is a target of two mitotic kinases, Cdk1p and Hrr25p.** *Traffic* 2007, **8:**647-660.
96. Loog M, Morgan DO: **Cyclin specificity in the phosphorylation of cyclin-dependent kinase substrates.** *Nature* 2005, **434:**104-108.
97. Holt LJ, Tuch BB, Villen J, Johnson AD, Gygi SP, Morgan DO: **Global analysis of Cdk1 substrate phosphorylation sites provides insights into evolution.** *Science* 2009, **325:**1682-1686.
